# Fully Characterized Mature Human iPS- and NMP-Derived Motor Neurons Thrive Without Neuroprotection in the Spinal Contusion Cavity

**DOI:** 10.3389/fncel.2021.725195

**Published:** 2022-01-03

**Authors:** Zachary T. Olmsted, Cinzia Stigliano, Brandon Marzullo, Jose Cibelli, Philip J. Horner, Janet L. Paluh

**Affiliations:** ^1^Nanobioscience Constellation, Colleges of Nanoscale Science and Engineering, State University of New York Polytechnic Institute, Albany, NY, United States; ^2^Center for Neuroregeneration, Department of Neurosurgery, Houston Methodist Research Institute, Houston, TX, United States; ^3^SUNY Buffalo Genomics and Bioinformatics Core, New York State Center of Excellence in Bioinformatics and Life Sciences, Buffalo, NY, United States; ^4^Department of Animal Science, College of Agriculture and Natural Resources, Michigan State University, East Lansing, MI, United States; ^5^Department of Large Animal Clinical Sciences, College of Veterinary Medicine, Michigan State University, East Lansing, MI, United States

**Keywords:** spinal cord injury, spinal motor neuron, neuromesodermal progenitor, oligodendrocyte progenitor cell, neuron transplantation, neuron survival, neuron maturation, contusion

## Abstract

Neural cell interventions in spinal cord injury (SCI) have focused predominantly on transplanted multipotent neural stem/progenitor cells (NSPCs) for animal research and clinical use due to limited information on survival of spinal neurons. However, transplanted NSPC fate is unpredictable and largely governed by injury-derived matrix and cytokine factors that are often gliogenic and inflammatory. Here, using a rat cervical hemicontusion model, we evaluate the survival and integration of hiPSC-derived spinal motor neurons (SMNs) and oligodendrocyte progenitor cells (OPCs). SMNs and OPCs were differentiated *in vitro* through a neuromesodermal progenitor stage to mimic the natural origin of the spinal cord. We demonstrate robust survival and engraftment without additional injury site modifiers or neuroprotective biomaterials. *Ex vivo* differentiated neurons achieve cervical spinal cord matched transcriptomic and proteomic profiles, meeting functional electrophysiology parameters prior to transplantation. These data establish an approach for *ex vivo* developmentally accurate neuronal fate specification and subsequent transplantation for a more streamlined and predictable outcome in neural cell-based therapies of SCI.

## Introduction

The feasibility to replace tissues damaged in spinal cord injury (SCI) *via* transplantation and to drive host-graft restored connectivity depends on optimizing therapeutic cell strategies. Central nervous system (CNS) cell replacement and regeneration initially used fetal tissue containing differently staged neural stem and progenitor cells (NSPCs), neurons and glia, and non-neuronal cell types (Reier et al., 1986; Bregman et al., 1993). Multipotent NSPCs generated *via* mouse and human stem cell neurotechnologies have now replaced this source in order to address the complexity of damage and loss of diverse CNS cell types in spinal networks after traumatic injuries (Silva et al., 2014; Assinck et al., 2017). Historically, there is no evidence that a mature neuron survives or thrives after transplantation. However, as stem cell neurotechnologies advance, breakthroughs in early human brain and trunk CNS development as well as RNA-Seq analysis are now enabling advanced neuronal differentiation strategies for generation of regionally-matched spinal neuron types and subtypes (Dulin et al., 2018; Olmsted and Paluh, 2021). Here we demonstrate that spinal motor neurons (SMNs) differentiated and matured *in vitro* from pluripotent stem cells can survive when transplanted into the SCI microenvironment in a 10 day and 6 week study.

A renewed interest to more directly address brain and trunk CNS pathologies with neurons is occurring in parallel with continuing NSPC efforts. Parkinson’s disease has seen great advancements by applying new stem cell neural differentiation technologies that provide anatomic regional matching and cell-type specific replacement (A9 dopaminergic neurons and their precursors; Kikuchi et al., 2017; Xiong et al., 2021). Pre-differentiated GABAergic spinal interneurons were recently tested for use in neuropathic pain models (Manion et al., 2020), although this study did not use an injury model and cells were injected into the intact dorsal horn. Due to a newly advanced understanding of human trunk and spine development (Henrique et al., 2015; Olmsted and Paluh, 2021; Wind et al., 2021), the spinal cord field is now poised to generate spinal neural cell types along the entire rostral-caudal neuraxis (Peljto et al., 2010; Kumamaru et al., 2018) and test these in a variety of animal SCI models. Developmentally directed neural cell types are expected to represent the gold standard for cell transplantation. The discovery of neuromesodermal progenitors (NMPs) as the multipotent building blocks of the human trunk and spine (Tzouanacou et al., 2009) revealed a novel pool of axial stem cells that give rise to the spinal cord neuroectoderm, mesodermal somites, and trunk neural crest cells (NCCs). The strategy of NMP-derived neural cells is gaining momentum as a possible optimized cell source for SCI therapeutics that is based in normal human progenitor development (Gouti et al., 2014) and could allow long-term survival in order to realize efficient host-graft interactions.

Stem cell pre-differentiated neurons for transplantation into traumatic models of SCI remain understudied but are emerging as a novel strategy in SCI cell therapeutics. Recently, a direct reprogramming approach bypassing the typical human developmental timeline was applied by Lee et al. (2020). In their acute transplantation study, de-differentiated autologous fibroblasts that were induced into motor neurons (iMNs) promoted functional recovery in a thoracic rodent spinal cord T9 compression model. Direct reprogramming of fibroblasts to iMNs vs. directed differentiation from pluripotent stem cells through developmental cascades results in inherent transcriptional differences with unknown long-term therapeutic impact (Burke et al., 2020; Carter et al., 2020) as well as cell variability that can impact reproducibility of SCI therapeutic outcomes. In this study, we differentiate spinal neurons from hiPSC-derived NMPs, which are yet to be evaluated in any postnatal mammalian SCI model, along with relevant spinal interneurons and oligodendrocyte progenitor cells (OPCs) in rat cervical hemicontusion SCI (C4-C5). By histological analysis at 10 days and 6 weeks, we show that NMP-derived homotypic neural cells delivered as suspensions survive in the harsh contusion injury microenvironment. Importantly, the SMNs survive without additional neuroprotective encapsulation or added facilitators, consistent with evidence that homotypic matching is a dominant factor in graft survival, integration and organization (Dulin et al., 2018). The ability to streamline cell therapeutics and achieve survival within the refractory lesion epicenter (Sontag et al., 2014), while bypassing the use of microenvironment regulators and/or encapsulation, is an important milestone for grafted neurons in SCI.

*Ex vivo* differentiation avoids the fate influence of the SCI microenvironment and gliogenic cues that are encountered *in vivo* by NSPCs, and instead employs more uniform differentiation to avoid variability in outcomes. We applied comprehensive analysis of SMNs by immunofluorescence imaging of biomarkers, RNA-Seq of temporally differentiating SMNs, and functional validation by co-culture assays with myotubes, phenotypic analyses of synaptic and ion channel repertoires, and functional microelectrode array (MEA) electrophysiology. Although the focus of this study is not oligodendroglia, we included hiPSC-derived OPCs that have been deemed necessary in other studies (Almad et al., 2011; Fan et al., 2017; Duncan et al., 2018). We assessed the microenvironment stability of homotypic SMNs and OPCs *in vivo* for SCI by transplanting co-cultures directly into the contusion cavity at the C4-C5 injury site, followed by immunohistochemistry of spinal cord sections 10 days and 6 weeks post-transplantation. Biomarker analysis revealed robust survival of the homotypic spinal neurons and OPCs, and rapid engraftment within the host tissue at both time points. We demonstrate emergent features of graft cell maturation over time such as OPC ramification and caudal axon navigation with anisotropic alignment in the host spinal cord. Transplanted OPCs lost progenitor biomarkers but did not yet obtain myelinating activity at 6 weeks, which classically occurs over a protracted time frame. This work is the first to assess unprotected NMP-derived homotypic SMNs and OPCs in a rodent model of SCI and demonstrates that these maturing cell types survive and consistently engraft within the subacute injury cavity. We propose that regional neural cell matching to the spinal cord will enable streamlining of SCI cell therapeutics to focus on neuronal connectivity and avoid variability introduced through *in vivo* NSPC reprogramming strategies.

## Materials and Methods

### Human iPSC Maintenance

The self-designated African American hiPSC line F3.5.2 was previously derived by reprogramming with Yamanaka factors in the Paluh and Cibelli labs and comprehensively characterized (Chang et al., 2015; Tomov et al., 2016). F3.5.2 hiPSC colonies were maintained in the pluripotency medium mTeSR Plus supplemented with 1× penicillin-streptomycin (P-S) on hESC-qualified Matrigel (1:100 dilution; Corning) at 37°C, 5% CO_2_. Cultures were passaged 1:6 in 6-well plates every 4–7 days depending on confluency using Gentle Cell Dissociation Reagent (GCDR, STEMCELL Technologies). Cells were stored at low passage number in mFreSR cryopreservation medium (STEMCELL Technologies). G-band karyotype was performed (Cell Line Genetics, Madison, WI) and cells were certified free of pathogens and mycoplasma.

### Lentivirus Transduction

GFP labeling of cells using lentivirus transduction was performed similarly to as described by Taylor et al. (2006) at the hiPSC pluripotent stage. Briefly, hiPSCs were seeded onto freshly coated 6-well plates 3 days prior to transduction with premade LV-CAG-eGFP lentivirus (Kerafast FCT149, 1 × 10^8^ CFU/ml). Polybrene (2 μg/ml; Millipore Sigma) was added to 2 ml mTeSR Plus. hiPSCs were transduced at 5 × multiplicity of infection (MOI) by centrifugation with the mTeSR-PB-LV medium for 1 h at 32°C, 1,200× *g* and subsequent incubation at 37°C, 5% CO_2_ for 18 h. Cultures were rinsed 2× with DMEM/F-12 and cultured in mTeSR Plus for 48 h prior to visual inspection by fluorescence microscopy. Transduced hiPSCs were selected and expanded in media with 2 μM puromycin. Cells were cryopreserved in mFreSR (STEMCELL Technologies) or further differentiated to OPCs described.

### Neural Differentiation and Specification to SMNs and OPCs

SMNs with caudal spinal cord identity were differentiated as previously described (Olmsted et al., 2020). Briefly, colonies at ~60–70% confluency were exposed to Neuromesodermal Progenitor Medium (NMPM). N2B27 basal medium: 1:1 DMEM/F-12:Neurobasal Plus medium, 2% (v/v) B-27 Plus supplement, 1% (v/v) N-2 supplement, 1× GlutaMAX, 1× MEM Non-Essential Amino Acids, 1× P-S (Gibco); NMPM: N2B27 supplemented with 40 ng/ml recombinant human (rh) FGF2 (R&D Systems), 40 ng/ml rhFGF8 (R&D Systems), 2 μM CHIR 99021 (Tocris Bioscience), 10 μM DAPT (Millipore Sigma), 10 μM SB431542 (Tocris Bioscience), 100 nM LDN193189 (Tocris Bioscience), and 0.36 U/ml heparin (Millipore Sigma). NMPM was changed daily. On day 5, NMPs were passaged using Gentle Cell Dissociation Reagent in Caudo-Ventral Patterning Medium (CVPM). CVPM: N2B27 supplemented with 100 nM Retinoic Acid (RA; Millipore Sigma), 200 nM Hh-Ag1.5. Y-27632 (Tocris Bioscience) was added at 10 μM during passages. Spinal cord NSC cultures were passaged 1:3 in 12-well plates and maintained at 90–100% confluency for 2–3 days before subsequent high-density passages (1:2). Neural cells were cultured on Matrigel for the entirety of differentiation. The NSCs were patterned in CVPM to day 25 and then transitioned to Terminal Differentiation Medium (TDM). TDM: N2B27 supplemented with 10 ng/ml rhBDNF, 10 ng/ml rhGDNF, 1 μM dibutyryl cyclic-AMP (dbcAMP; Millipore Sigma). Short-term 24–48 h neurosphere (NS) cultures were formed in 6-well plates (CELLTREAT) using a Scilogex MX-M microplate mixer at ~120 rpm and freshly seeded onto Matrigel substrate.

OPCs were differentiated through NMP and scNSC stages with the omission of DAPT during the first 5 days (Supplementary Figure 4A). On day 19 short-term 24 h NS suspension culture was used as a purification strategy. NS were seeded at day 20 in OPC medium (OPCM; Khazaei et al., 2017). This strategy specifies OPCs by biasing with growth factors IGF1, FGF2, and PDGF-AA in addition to the thyroid hormone triiodothyronine (T3) at day 20. OPCM: N2B27 (no Vitamin A) supplemented with 10 ng/ml rhIGF-1 (R&D Systems), 20 ng/ml rhFGF2 (R&D Systems), 20 ng/ml rhPDGF-AA (R&D Systems), 60 ng/ml tri-iodothyronine (T3; Millipore Sigma), and 0.36 U/ml heparin (Millipore Sigma). We validated OPCs by immunofluorescence (IF) of OLIG2/PAX6, Nkx-2.2, A2B5 and the OPC-specific antigen O4 (Supplementary Figure 4B).

### OPC Sorting and SMN-OPC Co-culture

We generated purified co-cultures of SMNs and OPCs prior to transplantation. The purification steps used were MACS separation of OPCs by the O4 antigen (Supplementary Figures 4C,D), and SMN-NS suspension culture and re-plating. Separated GFP-OPCs were mixed 1:2 with SMNs and co-cultured for in modified N2B27 medium for downstream SCI transplantation studies. GFP-OPCs were sorted manually using the MidiMACS kit (Miltenyi Biotec 130-090-312) with LS columns and Anti-PE MicroBeads (Miltenyi Biotec 130-048-801) according to manufacturer’s instructions. Cells were labeled with IgM PE-O4 antibody (R&D Systems FAB1326P) at 10 μl/10^6^ cells. GFP-OPCs were retained and mixed 1:2 with SMNs. Co-cultures were seeded and maintained for 1 week in modified N2B27 SMN-OPC medium prior to neural ribbon co-encapsulation. SMN-OPC Medium: N2B27 supplemented with 10 ng/ml rhBDNF, 10 ng/ml rhGDNF, 10 ng/ml rhNGF, 20 ng/ml rhPDGF-AA, 10 ng/ml rhIGF-1, 60 ng/ml T3, 100 μM dbcAMP, 25 μg/ml insulin, and 20 μg/ml ascorbic acid.

### Neuromuscular Junctions

Neuromuscular junction (NMJ) assays were performed similarly to as described in previous studies (Harper et al., 2004; Miles et al., 2004). Briefly, rat L6 myoblasts (ATCC CRL-1458) were seeded into Matrigel-coated Lab-Tek II 4-chambered cover glass and grown to confluency in DMEM/F-12, 10% FBS, 1× P-S medium. At 100% confluency, myoblasts were cultured in N2B27 medium supplemented with 100 nM RA, 200 nM Hh-Ag1.5 to drive multinucleated skeletal myotube formation. Twenty-four hours NS (day 38) were seeded onto myotubes at 5–8 NS per well in TDM and cultured for four days prior to fixation and fluorescence microscopy (day 42 SMNs).

### Phase Contrast Imaging and Immunofluorescence *In vitro*

Phase contrast images were acquired using a Zeiss Invertoskop 40C (5×/0.12 NA CP-Apochromat, 10×/0.25 NA Ph1 A-Plan and 20×/0.30 NA Ph1 LD A-Plan objectives; Olympus DP22 color camera). For immunofluorescence (IF), cells were seeded into Matrigel-coated glass-bottom chambers (Lab-Tek II 4-chambered cover glass; Nunc, #155382) and fixed with 10% buffered formalin for 30 min at appropriate stages in differentiation. Samples were permeabilized for 5 min in 0.1% Triton-X-100 and blocked for 30 min in 1% BSA fraction V (1× PBS). Primary antibodies were applied in 1 ml fresh blocking buffer and incubated at 4°C overnight (Supplementary Table 1, list of primary antibodies). Samples were rinsed thoroughly in 1× PBS before applying AlexaFluor secondary antibodies (Invitrogen) for 1 h in the dark with DAPI (4°C). Cells were imaged directly in chambered cover glass wells (Invitrogen). Fluorescence microscopy was performed using a Zeiss Axio Observer.Z1 inverted fluorescence microscope (20× /0.8 NA air and 63×/1.4 NA oil Plan-Apochromat DIC objectives). Images were acquired using an Hamamatsu ORCA ER CCD camera and Zeiss AxiovisionRel software (ver. 4.8.2). *Z*-stacks were gathered at 1 μm separation distance and compressed using the Extended Focus feature. Uniform exposure times were maintained across samples for identical antibodies. If necessary, images were adjusted linearly for brightness in Keynote or ImageJ.

### Calcium Imaging

SMN cultures were incubated at 37°C with 5 μM Fluo-4 AM (Invitrogen) for 30 min in DMEM/F12 (no supplements). Cultures were recovered in fresh medium for 30 min and imaged in HBSS using the Zeiss Axio Observer. Z1 system with the 20x air objective. Time lapse series were captured at an exposure time of 50 ms obtained in 200 ms intervals for a total duration of 1.5 min using the 488 nm LED. Frequency of transients and F/F_0_ over time was determined using ImageJ.

### Microelectrode Array Electrophysiology and Analyses

To monitor spontaneous neuronal activity in adherent SMN cultures, we seeded MNP-NS directly onto 60MEA200/30iR-Ti microelectrode arrays (MEA; Multi Channel Systems). MEA chips contained single wells with 60 electrodes per well, each of 30 μm diameter with 200 μm inter-electrode spacing (input impedance 30–50 kΩ). One electrode per MEA chip served as a reference electrode. To obtain recordings, we used an MEA2100 head stage system connected to an MCS-IFB-*in-vitro* interface board. Temperature was maintained at 37°C during recordings using a temperature controller. Arrays were coated with Poly-D-Lysine and 20 μg/ml Laminin. On day 28 of differentiation, 12–15 NS were seeded in TDM and allowed to adhere for 2 days, at which point one-half medium changes were made (1 ml total volume). One-half medium changes were subsequently made twice per week. Recordings were acquired after NS culturing for 6 days with Multi Channel Experimenter software (v2.14.0) sampled at 25 kHz for at least 5 min. Spike threshold for detection was automatically set to 5× above standard deviation over background noise (1 ms pre trigger, 2 ms post trigger). Burst detection parameters were set as follows: 50 ms interval to start and end burst, 100 ms minimum inter-burst interval, 50 ms minimum burst duration, four minimum spikes per burst. Spike and burst analyses were performed using Multi Channel Analyzer (v2.14.0) in conjunction with NeuroExplorer (v5.022), and are shown for days 35 and days 52 in differentiation in accordance with patch clamp experimental time points. For glutamate stimulation, we prepared a 10 mM solution in N2B27 medium and added glutamate to a final concentration of 50 μM. Recordings were taken for a minimum of 5 min and up to 14 min. *N* = 4–5 separate MEA chips were used for quantification per time point. Generation of raw data traces and statistical analyses were performed using GraphPad Prism 8 in conjunction with Microsoft Excel.

### Bulk RNA-Seq

Detailed information of RNA-Seq biological samples is provided in Supplementary Table 2. The datasets for SMN differentiated cell time points are also posted on NCBI GEO (GSE178600). SMNs were differentiated as described. RNA was extracted using the PureLink RNA Mini Kit (Invitrogen) according to manufacturer’s instructions. Neural cells from three wells of a 12-well plate were pooled for each sample for library preparation and RNA-Seq was performed at the SUNY Buffalo Genomics and Bioinformatics Core. Per-cycle basecall (BCL) files generated by the Illumina NextSeq were converted to per-read FASTQ files using bcl2fastq version 2.20.0.422 using default parameters and no lane splitting. The quality of the sequencing was reviewed using FastQC version 0.11.5 and FastqScreen version 0.11.1. Quality reports were summarized using MultiQC version 1.7 (Ewels et al., 2016). No adapter sequences were detected so no trimming was performed. Genomic alignments were performed using hisat2 version 2.1.0 using default parameters (Kim et al., 2015). The Illumina provided UCSC hg38 from their igenomes database was used for the reference genome and gene annotation set. Sequence alignments were compressed and sorted into binary alignment map (BAM) files using samtools version 1.7. Counting of mapped reads for genomic features was performed using Subread featureCounts version 1.6.2 using the parameters -p -s 2 –g gene_name -t exon -B -C -Q 60 the annotation file specified with –a was from UCSC hg38 (Liao et al., 2014). Alignment statistics and feature assignment statistics were again summarized using MultiQC. The unique alignment rate of the paired-end reads for all samples was around 85%. The number of mapped reads assigned to features ranged from about 8 million to 16 million. Differentially expressed genes were detected using the Bioconductor package DESeq2 version 1.24.0. DESeq2 tests for differential expression using a negative binomial generalized linear models, dispersion estimates, and logarithmic fold changes (Love et al., 2014). DESeq2 calculates log2 fold changes and Wald test *p*-values as well as preforming independent filtering and adjusts for multiple testing using the Benjamini-Hochberg procedure to control the false discovery rate (FDR). Histograms were generated using Microsoft Excel and GraphPad Prism.

### Cell Encapsulation, Overnight Shipping, and Cell Collection

Cells were encapsulated in neural ribbons for overnight shipping from Albany, NY to Houston, TX as previously described (Olmsted et al., 2020). Immediately after formation, neural ribbon quality was monitored by visual inspection. Shippable neural ribbons in 12-well plates were resuspended in N2B27 supplemented with 10 μM ROCK inhibitor, transferred to 1.8 ml cryovials using 1,000-μl pipette tips and immediately shipped on ice (4°C) *via* FedEx (20–22 h total in transit). At the destination laboratory, neural ribbons were recovered in a 37°C humidified cell culture incubator 1 day prior to transplantation. To collect cells for transplantation, alginate was rapidly dissolved in 1.6% sodium citrate solution and cells were pelleted by centrifugation and resuspended in HBSS supplemented with 20 ng/ml FGF2 and EGF and 5 mM glucose.

### Rat Cervical Spinal Cord Hemi-Contusion Injury and Transplants

A total of *N* = 6 rats were used in SCI grafting experiments. We performed cervical C4-C5 hemi-contusion injuries as previously described (Mondello et al., 2015). Briefly, injuries were made using the electromagnetic spinal cord injury device (ESCID). Right-sided hemi-laminectomy was performed at C4 level in anesthetized animals that were then placed in a spinal frame to clamp the lateral processes of C3, C5. The ESCID electromagnetic probe was positioned at the surface of the dura with an initial sensing force followed by rapid displacement (0.8 mm with 14 ms dwell). Afterwards, muscles were sutured in layers and the skin was clipped. The immunosuppressant tacrolimus was administrated 1 week prior transplantation of human cells at the dosage of 3.4 mg/kg/day through using a slow-release subcutaneous pellet implant (Innovative Research of America). Human SMNs and OPCs were shipped overnight as encapsulated neural ribbon constructs on ice (Olmsted et al., 2020), and received in Houston one day prior to injections. Constructs were suspended in fresh N2B27 and maintained in a humidified tissue culture incubator overnight. Cell number and retained viability was validated with an automatic counter (Invitrogen) before transplantation. For transplants, the spinal cord was re-exposed at injury level 15 days post-injury in anesthetized animals. To stabilize the animal, the lateral process of C3 was secured in a custom spinal frame. For delivery of high dose cell suspensions (*N* = 6 animals), alginate was dissolved using 1.6% sodium citrate and cells collected by centrifugation. Cells were delivered at ~200,000 cells per animal directly into the injury cavity by pulled glass pipette connected to Kopf micromanipulator in 2 μl of 5 mM glucose in HBSS vehicle with additional 20 ng/ml FGF and EGF for neurotrophic support. The glass pipette was retracted after delivery. The dura was sealed using a fibrin sealant made by mixing a 1:1 ratio of fibrinogen solution (20 mg/ml) with a bovine thrombin solution (10 U/ml; Millipore Sigma). Muscle and skin layers were closed, and animals received appropriate post-operative care. All rat experimental protocols were approved by Houston Methodist Research Institute IACUC and carried out in accordance with relevant ethical guidelines and regulations. This includes animal comfort, veterinary care, methods and reasons for euthanasia, and materials and hiPSC-derived neural injections.

### Tissue Processing and Immunohistochemistry

Spinal cords were harvested at two time points for short term (10 days post-transplantation, *N* = 3) and long term (6 weeks post-transplantation, *N* = 3) analyses. Animals were sedated with isoflurane and transcardially perfused first with ice cold 0.1 M PBS with 10,000 U of heparin. Tissues were then fixed by perfusion of 4% paraformaldehyde in 0.1 M PBS (pH 7.4). Spinal cords were harvested for further fixation in 4% paraformaldehyde at 4°C overnight. They were passed through a sucrose buffer gradient (10%, 20%, 30%; 24 h per solution) at 4°C for cryoprotection and aligned longitudinally in a Tissue Tek OCT block (Sakura, Nederland) using dry ice, and stored at −80°C. Forty micrometer thick spinal cord sagittal sections were prepared using a Cryostar NX50 cryomicrotome (ThermoFisher Scientific). Serial sections of the entire spinal cord were then mounted onto positively charged slides (ThermoFisher Scientific). For immunohistochemistry, slides were blocked with 10% goat serum in 0.2% Triton-PBS (T-PBS) for 1 h at room temperature followed by primary antibody incubation in 1% serum, 0.2% T-PBS overnight at 4°C. Primary antibodies used for *in vivo* studies were mouse anti-STEM121 (1:500; Takara, #Y40410, RRID: AB_2801314), chicken anti-GFAP (1:2,000; Abcam, #ab4674, RRID: AB_304558), rabbit anti-Synapsin 1 (1:200; Abcam, #ab64581, RRID: AB_1281135), rabbit anti-TUJ1 (1:1,000; BioLegend, #802001, RRID: AB_2564645), rabbit anti-OLIG2 (1:500; EMD-Millipore, #AB910, RRID: AB_2149918), mouse anti-O4 (1:500; R&D Systems, #MAB1326, RRID: AB_357617), mouse anti-APC (1:50; EMD-Millipore, #OP80, RRID: AB_205371). GFP-OPCs retained GFP signal after fixation. After three additional wash steps with 1% serum T-PBS, slides were incubated with secondary antibodies in PBS for 1 h. Secondary antibodies used were goat anti-rabbit IgG Alexa Fluor (AF) 568 (ThermoFisher Scientific, #A-11011, RRID: AB_143157), goat anti-mouse IgM AF 647 (ThermoFisher Scientific, #A-21238, RRID: AB_2535807), goat anti-mouse IgG2b AF 647 (ThermoFisher Scientific, #A-21242, RRID: AB_2535811), goat anti-mouse IgG1 AF 568 (ThermoFisher Scientific, #A-21124, RRID: AB_2535766), goat anti-chicken IgY Cy5 (ThermoFisher Scientific, #A-21449). Secondaries were added at a dilution of 1:1,000. Slides were mounted with DAPI-Permount (ThermoFisher Scientific) and imaged with a Leica DMi8 confocal microscope.

Graft volume and area at the lesion site were calculated with the stereological probe Cavalieri using the software StereoInvestigator (MBF Bioscience). Specifically, the lesion area of the cord (4 mm), cavity, and graft size were determined on every five sections by tracing the different areas in the cords transplanted with cell suspensions and sacrificed at 6 weeks (*N* = 3). Transplant area was defined in STEM121/DAPI longitudinal sections 40 μm thick using a Zeiss Axioskop 2 microscope. For the Cavalieri method, the following parameters were chosen: grid size (μm): 100; Associated Area (μm^2^): 10,000; Section Cut Thickness (μm): 40; Section Evaluation Interval: five; Associated Volume (μm^3^): 2,000,000; Number of Sections: four. The number of GFP-OPCs and GFP-OPCs/GFAP+ were estimated with stereology analysis using the Optical Fractionator probe in StereoInvestigator software and an Axioskop2plus Zeiss microscope. For further analysis, the percentage of GFP-OPCs/GFAP+ cells was calculated by dividing estimated population number of GFP-OPCs/GFAP+ by the estimated population total number of GFP-OPCs. Data are provided as averages of estimated population of cells using defined section thickness of three rats (6 weeks time point), five sections per rat with 40 μm section cut thickness.

### Experimental Design and Statistical Analyses

Raw data were compiled in Microsoft Excel (v16.16.16) and exported to GraphPad Prism (v8.3.0) for plotting and statistical analyses. Data are reported as mean ± s.e.m.) and analyzed using unpaired two-tailed t-test unless otherwise specified. Cells were manually counted in ImageJ to quantify immunofluorescence data. ****p < 0.0001 ***p < 0.001 **p < 0.01 *p < 0.05 n.s. not significant (*α* = 0.05). Power analysis was not performed. Detailed information for each experiment is provided in figure legends.

### Figures

Figures for this manuscript were made in Keynote (v9.2.1), Canvas Draw (v4.0.1), Adobe Illustrator CC (v25.0.1), and BioRender.com. Data plots were generated using GraphPad Prism (v9) or Microsoft Excel (v16.16.19).

## Results

### Generation of SMNs From hiPSCs Using Trunk-Biased Neuromesodermal Progenitors

**Figure 1:**
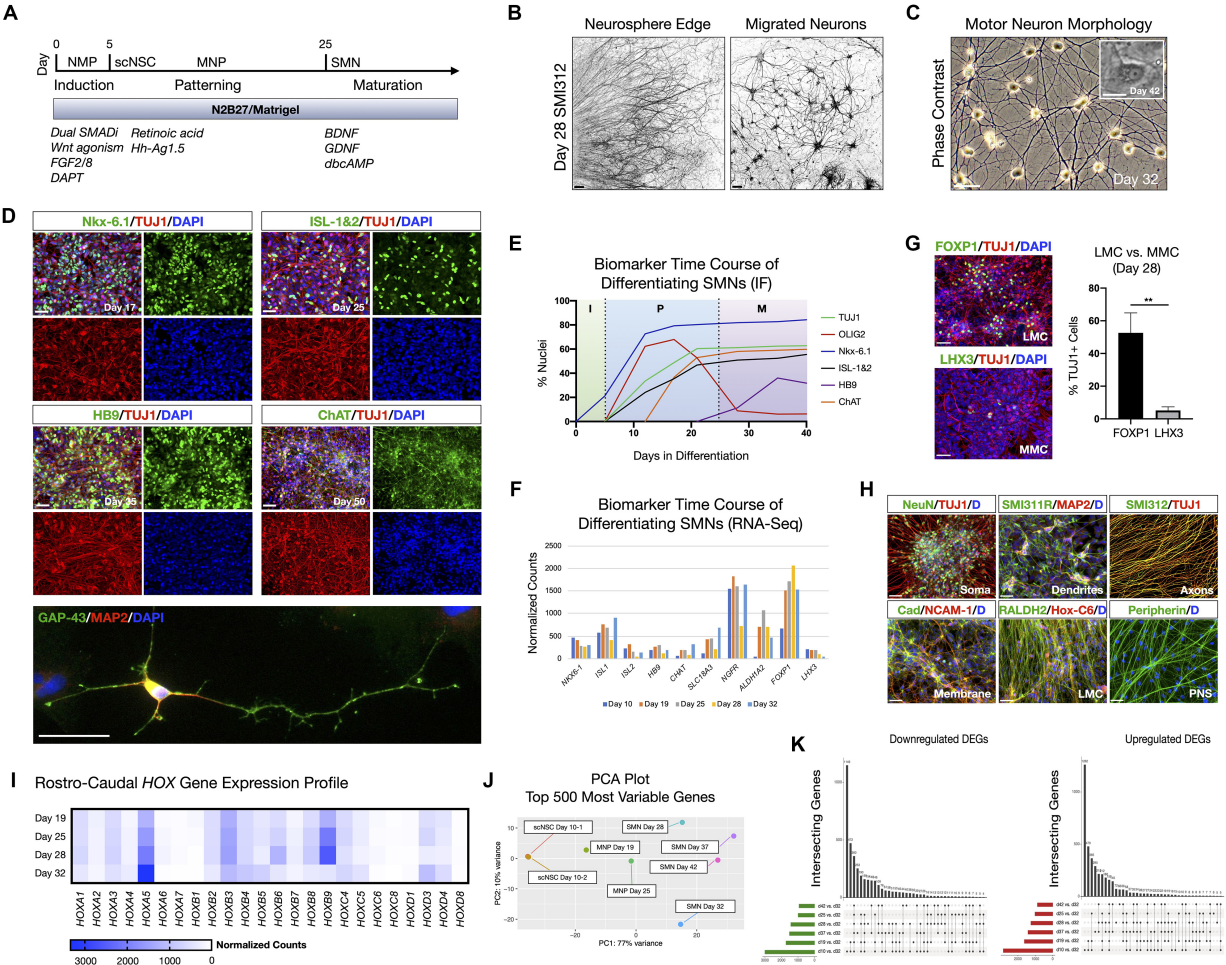
hiPSC-derived scNSCs generate SMNs regionally-matched for the spinal cord. **(A)** Overview of differentiation protocol used to generate cervically-patterned SMNs through NMP, scNSC, and MNP stages. Small molecules and growth factors used in caudo-ventral patterning and maturation are listed. **(B)** Pan-axonal antibody SMI312 labeling of day 28 adherent neurosphere (NS) culture at NS edge (left) and more distal field of migrated neurons (right). SMI312 signal shown with inverted LUT. **(C)** Phase contrast images of day 32 SMN cultures. Inset is day 42 single SMN with characteristic trapezoidal shape and prominent nucleolus. **(D)** SMN hallmark biomarkers. Top: Motor progenitor spinal domain transcription factor Nkx-6.1 (day 17); SMN-related factors ISL-1&2 (day 25); Middle: SMN-specific HB9 (day 35); choline acetyltransferase enzyme ChAT (day 50); Cells are counterstained with TUJ1 and DAPI. Bottom: IF of single neuron. GAP-43/MAP2/DAPI (day 32). **(E)** Time course of biomarker expression in differentiating SMN cultures (I, induction; P, patterning; M, maturation). Percent of nuclei were counted from IF images. *N* = 3 fields were averaged per biomarker per time point (>100 cells per field). **(F)** Time course biomarker expression of differentiating SMN cultures by bulk RNA-Seq. **(G)** IF images and histogram of later motor column (LMC) SMN transcription factor FOXP1 (53 ± 12%; *n* = 349 cells) vs. medial motor column (MMC) SMN transcription factor LHX3 (5 ± 2%; *n* = 304 cells; two-tailed t test, *t* = 6.629, df = 4, **P = 0.0027). **(H)** Visualizing neuronal compartments and SMN regional specificity by biomarker. **(I)**, RNA-Seq validation of cervical spinal cord identity by normalized Hox gene expression profile in differentiating SMN cultures. **(J)** PCA plot of 500 most variable genes during scNSC neuronal differentiation. **(K)** UpSet plots of intersecting downregulated (left) and upregulated (right) differentially expressed genes (DEGs) using individual time point comparisons in differentiation. Plot height represents the number of overlapping DEGs between the sets below marked with filled circles. Vertical lines connecting two or more dots denote the set of genes overlaps the connected sets. Data are reported as mean ± s.e.m. Scale bars are 50 μm, and 10 μm in **(C)** inset.

To generate neurons appropriate for cervical SCI, spinal motor neurons (SMNs) were derived in three stages (Figure 1; Supplementary Figure 1), referred to here as: (1) neural induction; (2) caudo-ventral patterning; and (3) neuronal maturation (Figure 1A). Before neural induction, we first differentiated the hiPSC line F3.5.2 to neuromesodermal progenitors (NMPs) that are bipotent intermediates preceding spinal cord neuroectodermal development (Chang et al., 2015; Lippmann et al., 2015; Tomov et al., 2016). We previously showed that this protocol generates SOX2/Bra NMPs at high efficiency (Olmsted et al., 2020, 2021). Synergistic Wnt/β-catenin and FGF-mediated signaling pathways were stimulated using the small molecule CHIR 99021 and recombinant human FGF2/FGF8 proteins, respectively, in the context of dual SMAD inhibition with LDN 193189 and SB 431542 (Chambers et al., 2009, 2012) during the first 5 days of differentiation. The GSK-3β inhibitor DAPT was also included in the growth medium during this time. NMP transition to spinal cord NSC rosettes (scNSCs) was triggered by switching to caudo-ventral patterning medium containing 100 nM of the caudalizing morphogen, retinoic acid (RA), that promotes neuralization and 200 nM of the ventralizing morphogen, Hh-Ag1.5, a potent agonist of Sonic hedgehog (Supplementary Figure 1A). Continued exposure yields robust motor neuron progenitor (MNP) cultures by day 12 that were immunopositive for Nestin, OLIG2, TUJ1, and NeuN (Supplementary Figures 1B–D). Differentiating SMNs were validated using bulk RNA-Seq at seven time points including days 10, 19, 25, 28, 32, 37, and 42 (Supplementary Figures 1E,F). Day 32 cultures had an expression profile that was most indicative of desired SMNs (e.g., HB9/FOXP1/ISL1/PRPH/STMN2/VGF/CHAT/MAP2/TUBB3) and so we compared all other datasets to this stage individually, shown with volcano plots (Supplementary Figure 1G). Day 32 SMNs have perinuclear and polarized expression of STMN2 in GAP-43 positive neurites, also typical of developing SMNs (Supplementary Figure 1H, Klim et al., 2019).

We used short-term (24–48 h) neurosphere (NS) suspension cultures as a purification step prior to neuronal maturation. Plated NS cultures projected aligned axons radially outward along which neurons migrated to form distal 2D adherent regions, visualized by the pan-axonal marker SMI312 (Figure 1B). At day 25, cultures were transitioned to and subsequently maintained in terminal differentiation medium containing 10 ng/ml BDNF, GDNF, and 1 μM dbcAMP. Adherent neurons exhibited typical SMN morphology as assessed by trapezoidal soma shape, a prominent single nucleolus, and dendritic arborization (Figure 1C). The SMNs expressed a panel of characteristic biomarkers by immunofluorescence (IF) time course (Figures 1D,E). That is, the ventral motor neuron progenitor domain transcription factor NK6 homeobox 1 (Nkx-6.1), SMN-related ISL LIM homeobox 1 and 2 (ISL-1&2), SMN-specific homeobox 9 (HB9/*MNX1*), and choline acetyltransferase (ChAT). Growth associated protein 43 (GAP-43) localized to neuronal growth cone and branching structures (Figure 1D). By developmentally relevant differentiation cues, we generated the appropriate cervical SMNs through neuromesodermal progenitors reflecting characteristic morphological and biomarker expression.

### Developmental Hallmarks of NMP-Derived Spinal Motor Neurons Validated By RNA-Seq, Biomarkers, and Functional Assays

SMNs derived through NMP intermediates were further validated by whole-transcriptome bulk RNA-Seq and IF on a differentiation time course (Figure 1; Supplementary Figure 1). SMNs primarily expressed lateral motor column (LMC) transcription factor FOXP1 (53 ± 12%, *n* = 349 cells) vs. the medial motor column (MMC) transcription factor LHX3 (5 ± 2%, *n* = 304 cells) that was downregulated over time (Figures 1F,G; Amoroso et al., 2013). We verified general biomarker expression for neuronal compartments that are somatic (NeuN), dendritic (SMI311R/MAP2), axonal (SMI312) and membrane (Pan-Cadherin/NCAM-1), and phenotypic regional specificity to the LMC (Hox-C6, RALDH2, Peripherin) by IF (Figure 1H). Caudal cervical spinal cord identity is consistent with collinear *Hox* gene expression profiles using bulk RNA-Seq (Figure 1I). Principle component analysis (PCA) plots of the top 500 most variable genes revealed the similarities between scNSC biological replicates, but a progressive dissimilarity between time points in longer differentiated cultures (Figure 1J). UpSet plots for downregulated [log_2_(fold change) <0] and upregulated [log_2_(fold change) >0] differentially expressed genes (DEGs; *P*_adj_ < 0.05) enable visualization of multiple set intersections similarly to Venn or Euler diagrams (Figure 1K). However, unlike Venn or Euler diagrams, UpSet scales appropriately when comparing three or more groups and also maintains correct intersectional area proportions. Differentiating samples were compared to day 32 SMNs and the trend was the same for both downregulated and upregulated DEGs.

**Figure 2:**
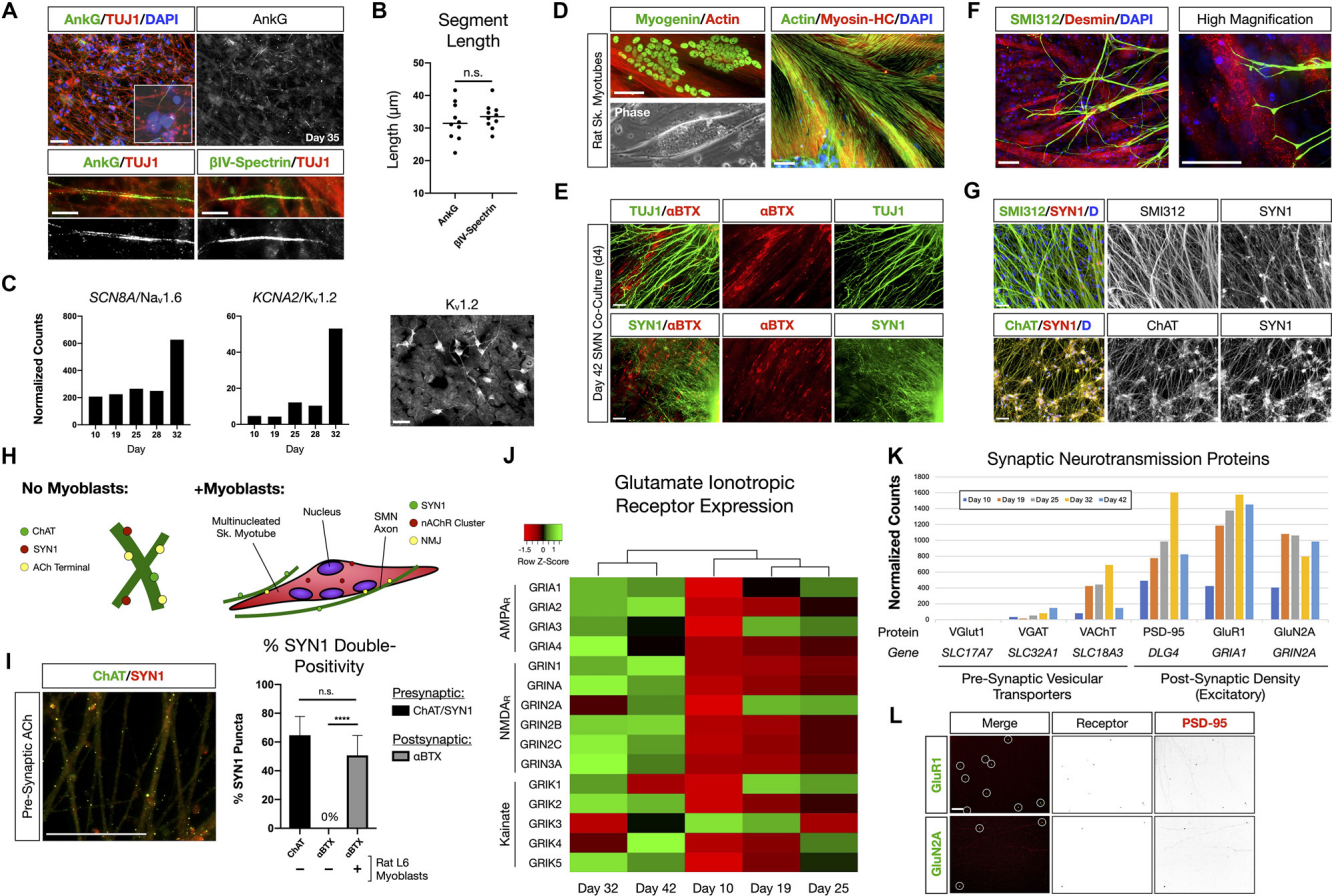
NMP-derived SMNs are positive for axon initial segment proteins, assemble neuromuscular junctions, and develop characteristic synaptic profiles. **(A)** Maturing SMN-NS cultures exhibit short segments immunopositive for AIS proteins Ankyrin-G (AnkG) and βIV-Spectrin at day 35. Top: AnkG/TUJ1 counterstained with DAPI (nuclei). High-magnification image depicts AnkG+ staining distal to the soma. Bottom-left: AnkG immunopositive region along TUJ1+ filaments. Bottom-right: βIV-Spectrin immunopositive region along TUJ1+ filaments. **(B)** Length of AnkG (32 ± 2 μm) and βIV-Spectrin (34 ± 1 μm) segments by day 35 (two-tailed t test, *t* = 0.7082, df = 18, *P* = 0.4879, *n* = 10 segments measured per antibody). **(C)** Time course normalized expression of AIS-related voltage gated ion channels Na_v_1.6 and K_v_1.2 by RNA-Seq. IF showing cells positive for K_v_1.2 on right. **(D)** Rat L6 multinucleated skeletal myotubes for NMJ formation assays. **(E)** Day 4 co-culture of SMN-NS (day 42) seeded onto rat myotubes. Top: TUJ1+ filament termination onto αBTX+ junctions (nAChR). Bottom: SYN1/αBTX. **(F)** SMI312 axon termination in Desmin+ myotubes resembling early motor end plates. **(G)** Co-localization of cholinergic terminals (ChAT, bottom) with SYN1 puncta along axons (SMI312, top). **(H)** Cartoon diagram of ChAT/SYN1 pre-synaptic ACh terminals and nAChR post-synaptic machinery. **(I)** IF image and histogram of ChAT/SYN1 (64.7 ± 13.1%, *n* = 118 puncta) and SYN1/αBTX co-localization in the absence (0%, *n* = 48 puncta) or presence (50.7 ± 13.8%, *n* = 69 puncta) of L6 myotubes (two-tailed t test, *t* = 10.36, df = 14, ****P < 0.0001). *N* = 8 fields were averaged for each condition. **(J)** Heatmap of glutamate ionotropic receptor expression (AMPA_R_, NMDA_R_, Kainate) during SMN differentiation. **(K)** Histogram of normalized gene expression for pre-synaptic vesicular transporters (VGlut1 excitatory, VGAT inhibitory, VAChT cholinergic) and post-synaptic density proteins/excitatory receptors. **(L)** Co-localization of AMPA_R_ GluR1 (top) and NMDA_R_ GluN2A (bottom) with scaffolding protein PSD-95. Individual channels provided as inverted LUT. Data are reported as mean ± s.e.m. Scale bars are 50 μm, and 10 μm in **(A)** high magnification images (bottom). n.s., not significant.

We further interrogated phenotypic maturation and functional hallmarks of the SMNs using numerous assays (Figure 2). At day 35 in differentiation, SMNs were immunopositive for neuronal markers of the axon for axon initial segment (AIS) proteins Ankyrin-G (AnkG) and βIV-Spectrin (Figure 2A). As expected, immunostaining occurred as short segments along TUJ1 labeled microtubule neuronal filaments distal to the soma for both AnkG (32 ± 2 μm) and βIV-Spectrin (34 ± 1 μm; mean ± s.e.m.; Figure 2B). Maturation of signaling potential was reflected in time course RNA-Seq analysis of genes encoding the voltage-gated ion channels Na_v_1.6 (*SCN8A*) and K_v_1.2 (*KCNA2*) that additionally supports ongoing neuronal maturation in these cultures (Figure 2C).

The ability to assemble neuromuscular junction (NMJ) machinery was investigated between species by co-culturing hiPSC-derived SMNs with multinucleated skeletal myotubes formed from rat L6 myoblasts (Figure 2D). Co-culture of SMN-derived NS (day 38) with rat myotubes for 4 days resulted in marked co-localization of TUJ1 and Synapsin 1 (SYN1) neuronal filaments with α-bungarotoxin (αBTX), a high-affinity binding antagonist of nicotinic acetylcholine receptors (nAChR) that form the post-synaptic junction of NMJs (Figure 2E). SMN axons identified with SMI312 labeling terminated abruptly onto myotubes and formed structures resembling early motor end plates (Figure 2F). The NMJ presynaptic terminal contains a network of ion channels including those for release of acetylcholine (ACh). We further observed co-localization of the choline acetyltransferase enzyme, ChAT, with SYN1 puncta along SMN axons, identifying the cholinergic pre-synaptic terminals (64.7 ± 13.1%, *n* = 118 SYN1 puncta; Figures 2G–I). Post-synaptic αBTX did not co-localize with SYN1 puncta in control SMN mono-cultures. Thus our SMNs reflect multiple functional hallmarks of NMJs.

SMN activity in the spinal cord is influenced by descending cranial motoneuron (MN) fibers *via* glutamatergic neurotransmission. We therefore also investigated the expression of glutamate ionotropic receptors by the SMNs (Figures 2J–L). Clustering analysis of RNA-Seq data revealed that SMNs cluster separately from scNSCs and MN progenitors with progressive activation of mRNA expression for genes encoding AMPA, NMDA and Kainate receptors from stages of scNSCs through to SMNs as a general trend (Figure 2J). In accordance with observed immunostaining and pre-synaptic co-localization of ChAT and SYN1 in SMN NMJs, RNA-Seq data reveals that the SMNs primarily expressed the acetylcholine vesicular transporter VAChT vs. inhibitory (VGAT) or excitatory (VGlut1) vesicular transporters. The post-synaptic density scaffolding protein PSD-95 (*DLG4*) was highly expressed along with excitatory receptors, AMPA receptor subunit GluR1 (*GRIA1*) and the NMDA receptor subunit GluN2A (*GRIN2A*; Figure 2K) and these are co-localized by immunostaining (Figure 2L). In summary, the RNA-Seq data along with biomarker immunostaining reflect the maturation of SMNs *in vitro* through analysis of pan-neuronal and SMN specific biomarkers, and formation of functionally specific NMJ structures.

### Characterization of SMN Ventral Patterning Efficiency and Gliogenesis in scNSC-Differentiated SMNs

Ventral and dorsal spinal interneurons (INs) along with astrocytes that promote synaptogenesis are indispensable to normal neuronal circuit function *in vivo*. We previously investigated the identities and proportions of spinal interneurons generated using the NMP-derived, SMN-directed differentiation protocol by immunofluorescence (Olmsted et al., 2021). Here we investigated ventral vs. dorsal identity and the presence of glia in our differentiating neuronal cultures by RNA-Seq. As expected from our differentiation protocol, the cultures primarily exhibited ventral rather than dorsal spinal cord identity by RNA-Seq normalized counts of characteristic progenitor domain and neuronal subtype transcription factors (Supplementary Figure 2A, Sagner and Briscoe, 2019). Genes for oligodendroglial and astroglial lineages were also identified in differentiating SMN-directed cultures (Supplementary Figures 2B,C), although we refer to the SMN cultures as simply “SMNs” throughout the text. GFAP+ astrocytes were not observed until day 40 in differentiation, consistent with late waves of gliogenesis subsequent to an initial neurogenesis phase in human gestation. The day 32 neurons that we transplant prior to this differentiation time point did not contain GFAP+ astrocytes and therefore are expected to rely on host astrocytes for metabolism and sustained viability.

### SMNs Develop Regular Spiking Patterns and Glutamate-Responsive Bursting

**Figure 3:**
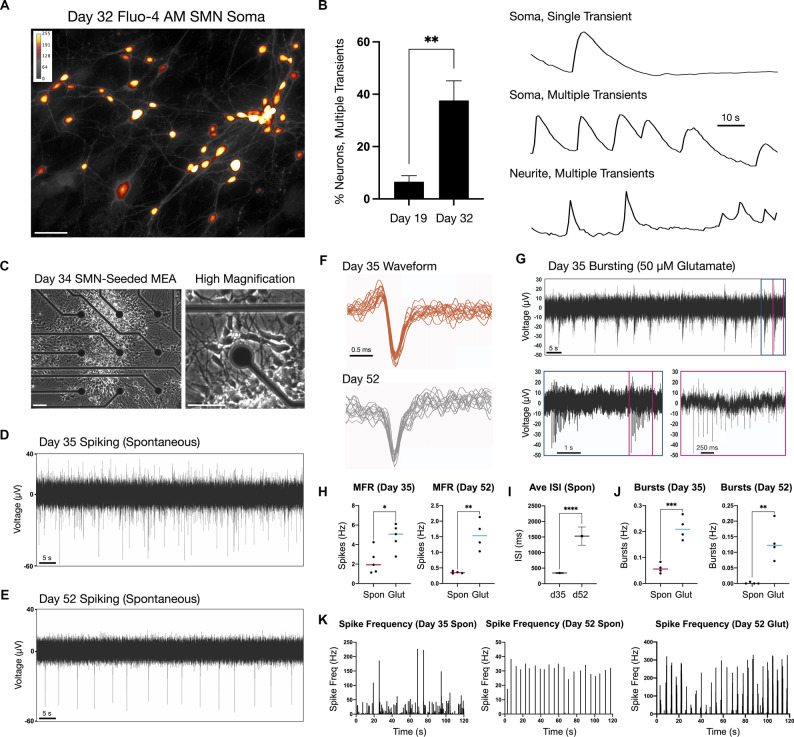
Firing properties of adherent spinal neurons by calcium imaging and microelectrode arrays (MEA) at two time points. **(A)** Calcium dye Fluo-4 AM somatic signal in adherent SMNs (day 32). ImageJ Smart LUT fluorescence intensity (yellow high, gray low). **(B)** Left: Proportion of SMNs exhibiting multiple calcium transients above threshold in the 1.5 min recording window at day 19 (6.6 ± 2.3%, *n* = 83 neurons, *N* = 5 repeat experiments) and day 32 (37.6 ± 7.5%; *n* = 101 neurons, *N* = 5 repeat experiments). **P = 0.0043. Right: Representative examples of single and multiple transients in soma and neurite of day 32 SMNs over the 1.5 min recording window (F/Fo). Scale bar is 10 s. **(C)** Phase contrast images of SMN-seeded MEAs at day 34 in differentiation (seeded day 28). **(D,E)** Representative traces from single MEA channel depicting spontaneous SMN voltage spikes recorded at day 35 **(D)** and day 52 **(E)** in differentiation. **(F)** Representative overlay of spontaneous spike waveforms at day 35 and day 52 from traces in **(D,E)**. **(G)** Representative single electrode bursting in day 35 cultures stimulated with 50 μM glutamate. Three time scales are shown. Pink box depicts spikes within a single burst. **(H)** Quantification of mean firing rate (MFR, Hz) in day 35 and day 52 cultures. Spontaneous firing is compared to 50 μM glutamate stimulation (two-tailed *t* test; day 32 *t* = 3.096, df = 8, *P = 0.0148; day 52 *t* = 4.968, df = 6, **P = 0.0025). **(I)** Average inter-spike interval (ISI) in day 35 vs. day 52 SMNs under spontaneous conditions (*t* = 10.52, df = 499, ****P < 0.0001). **(J)** Single electrode burst rate (Hz) in day 35 vs. day 52 SMNs (day 32 *t* = 6.426, df = 6, ***P = 0.0007; day 52 *t* = 4.356, df = 6, **P = 0.0048). **(K)** Spike frequency (Hz) vs. time (s) in day 35 vs. day 52 SMNs. Also shown is spike frequency for day 52 SMNs stimulated with 50 μM glutamate is shown (rightmost plot). Spontaneous (spon), glutamate (glut). Scale bars are 50 μm.

Neuronal firing was examined by calcium imaging as a proxy in addition to MEA extracellular recordings (Figure 3). We measured calcium transients in the soma and neurites of day 19 and 32 adherent SMNs using the cell-permeant calcium sensitive dye, Fluo-4 AM (Figures 3A,B). Cultures were imaged in HBSS medium without additional chemical stimulation (e.g., glutamate) and calcium transients quantified. At day 19, 6.6 ± 2.3% of SMNs imaged exhibited multiple calcium transients above threshold during the 1.5 min recording window as measured in the soma (*n* = 83 neurons, *N* = 5 repeat experiments). This increased to 37.6 ± 7.5% of SMNs at day 32 (*n* = 101 neurons, *N* = 5 repeat experiments, **P = 0.0043; Figure 3B, left). Representative examples of single and multiple transients in soma and neurite are provided (F/Fo; Figure 3B, right). Mean rate of calcium transients as measured in the soma was 6.6 ± 1.2 transients/min at day 32.

For MEA analysis, we seeded day 28 neurospheres (NS) onto single 60-electrode arrays (12–15 NS/array) in terminal differentiation medium (Figure 3C; day 34) and maintained adherent cultures to day 52. The firing activity recorded using MEAs is described for day 35 and day 52 time points (Figures 3D–K). Day 35 cultures exhibited high and irregular spontaneous firing (Figures 3D–H). By contrast, day 52 cultures displayed lower and more regular spontaneous firing rates (Figures 3E–H; mean firing rate, MFR; Figures 3I–K). The RNA-Seq data analysis of the SMNs reveals they have the receptor machinery to respond to glutamate. Notably, in both day 35 and in day 52 cultures, addition of 50 μM glutamate to the extracellular medium increased neuronal firing and produced robust bursting activity (day 35 ***P = 0.0007, day 52 **P = 0.0048). Glutamate-stimulated bursting was more pronounced and became qualitatively rhythmic in day 52 SMNs, and this effect persisted for the entire duration of the experiment (Figure 3K). Network bursts were not detected during the time of recordings using 2D cultures. To supplement electrophysiological characterization, we also examined a differentiation time series of potassium and sodium ion channel gene expression known to be critical for neuronal function and maturation (Supplementary Figures 3A,B). Our findings reflect functional maturation of spiking activity over time advanced, but was not enhanced by substantially longer culturing times (day 52) beyond day 32 SMNs. This is consistent with our previous studies in which the three-dimensional context of cells seems to play a greater role (Olmsted et al., 2021).

### Transplanted Homotypic SMN-OPCs Survive Within the Contused Injury Cavity and Demonstrate Long-Term Histological Evidence of Integration

**Figure 4:**
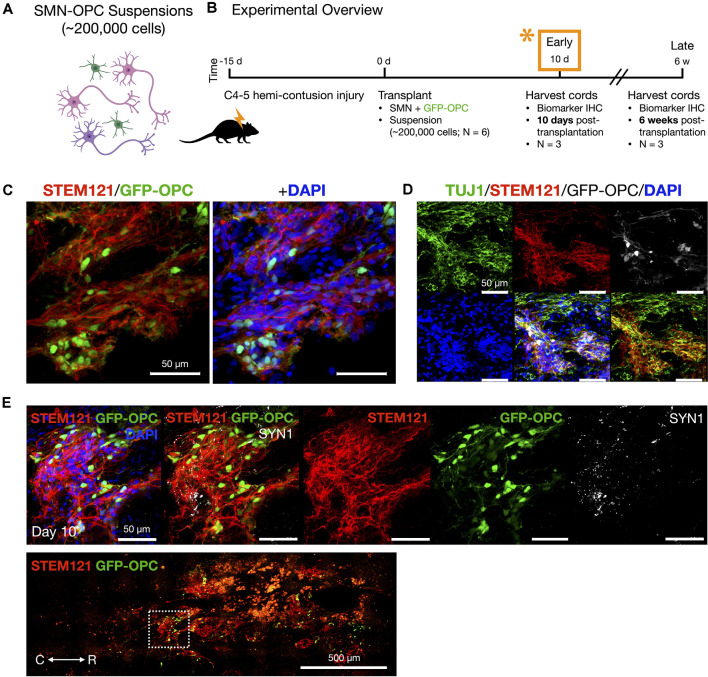
*In vivo* delivery of SMN-OPC cell suspensions into the contusion cavity and survival at 10 days post-transplantation. **(A)** Schematic overview of the SMN-OPC cell suspension grafts. Pink depicts spinal motor neurons, purple depicts spinal interneurons that co-differentiate, and green is GFP-OPCs. **(B)** Schematic overview of SMN-OPC cervical hemicontusion grafting experiment (*N* = 6 animals). *Data provided in this figure are from the 10 days time point. **(C)** STEM121/GFP-OPC/DAPI merged image at the 10 days time point. **(D)** TUJ1 immunostain validates retention of the neuronal phenotype since this antibody detects the neuron-specific β-tubulin isotype, β-III-tubulin. **(E)** High magnification image of suspension SMN and GFP-OPC survival 10 days post-transplantation. SYN1 co-localization with STEM121 is shown. Low magnification STEM121/GFP-OPC image denotes position of field acquired caudal to the lesion epicenter (white box). R (rostral), C (caudal). Individual scale bars are provided.

**Figure 5:**
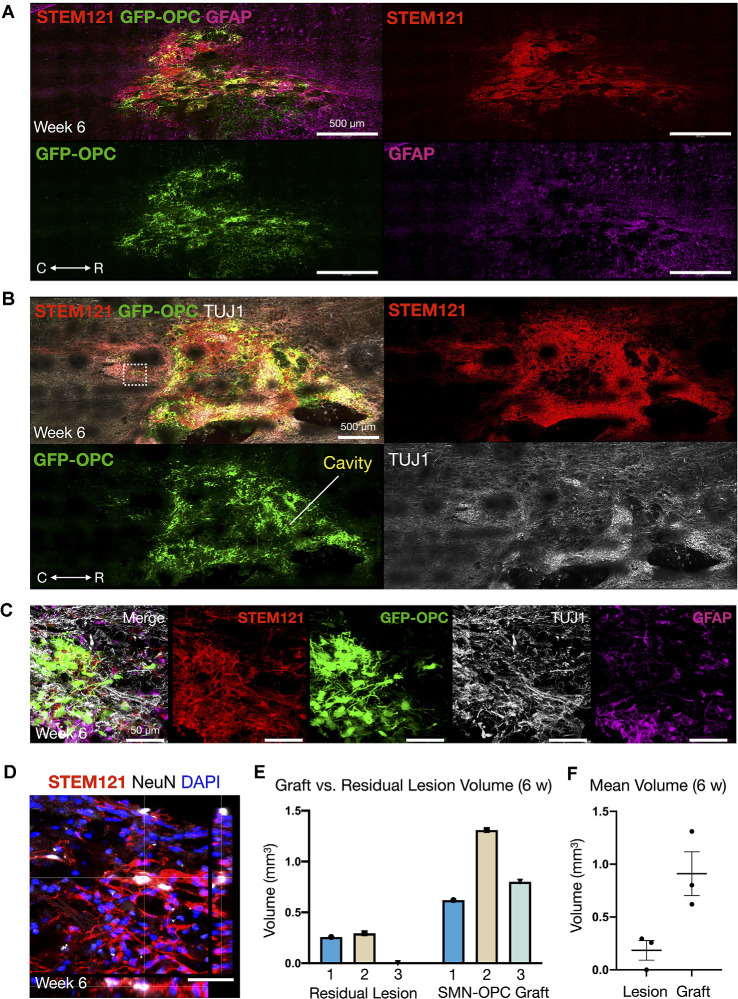
Continued survival of SMN-OPC cell suspensions at 6 weeks post-transplantation. **(A)** Low power image of the graft at 6 weeks shown with STEM121/GFP-OPC/GFAP to depict host glial cells. **(B)** Low power image of the graft at 6 weeks shown with STEM121/GFP-OPC and TUJ1 to validate retention of the neuronal phenotype. White box denotes high magnification field shown in **(C)**, imaged caudal to the lesion epicenter. **(C)** High magnification merged image and individual channels (STEM121/GFP-OPC/TUJ1/GFAP) at 6 weeks post-transplantation. **(D)** STEM121 immunostain with mature neuronal marker NeuN co-localization. **(E,F)** Cavalieri volumetric analysis of the residual lesion and grafts. Color matching corresponds to the same animal. Shown are residual lesion vs. graft volume for individual spinal cords at 6 weeks (mm^3^) **(E)** and mean ± s.e.m **(F)**. *N* = 3 animals were sacrificed at 6 weeks post-transplantation. R (rostral), C (caudal). Individual scale bars are provided.

**Figure 6:**
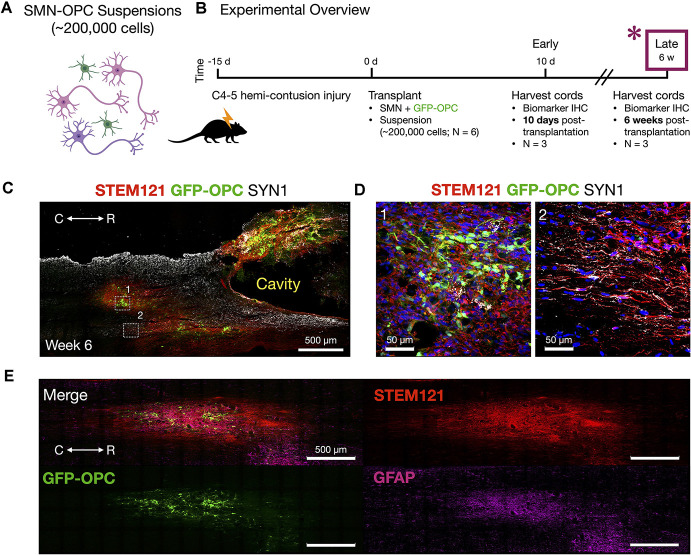
Host tissue contusion site integration of SMN-OPC *in vivo* grafts. **(A)** Schematic overview of the SMN-OPC cell suspension grafts. Pink depicts spinal motor neurons, purple depicts spinal interneurons that co-differentiate, and green is GFP-OPCs. **(B)** Schematic overview of SMN-OPC cervical hemicontusion grafting experiment (*N* = 6 animals). *Data provided in this figure are from the 6 weeks time point. **(C)** Low magnification view of SMN-OPC suspension graft using STEM121 and SYN1 antibodies, and GFP to identify OPCs. White boxes (1, 2) denote high magnification fields shown in **(D)**. **(D)** Top: high magnification images of 6 weeks suspension grafts in the host spared spinal cord (1, 2) visualized with STEM121/GFP-OPC/SYN1/DAPI. Neuronal projections into spared host tissue have robust co-localization with SYN1. **(E)** Merged image and individual channels (STEM121/GFP-OPC/GFAP) demonstrates filling of the injury cavity and aligned fiber outgrowth in the host spared tissue. R (rostral), C (caudal). Individual scale bars are provided.

**Figure 7:**
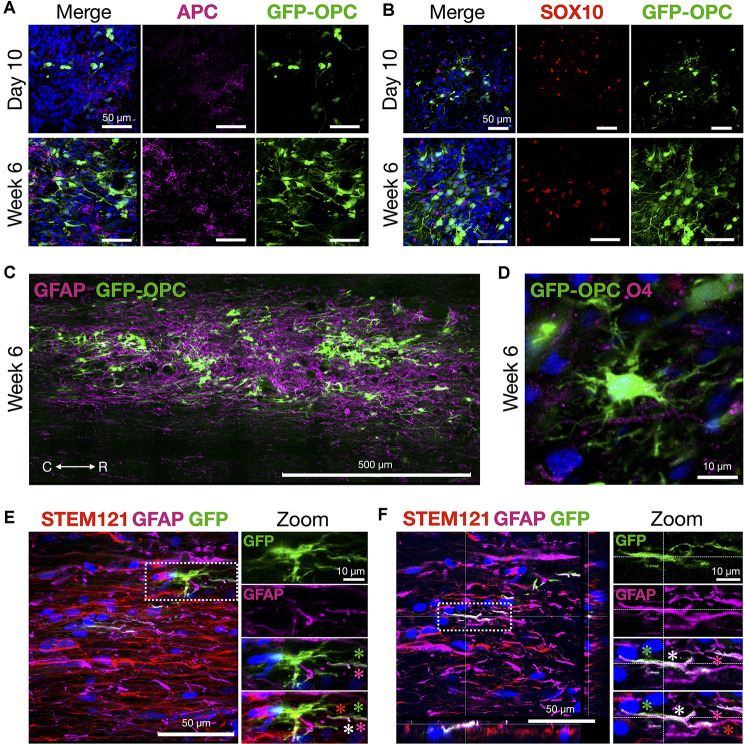
OPCs thrive and morphologically develop in the contusion injury site. **(A)** Mature oligodendrocyte marker APC and transplanted GFP-OPCs at 10 days and 6 weeks time points, counterstained with DAPI. **(B)** GFP-OPCs increase in morphological complexity over 6 weeks in association with SOX10+ cells in the contusion injury microenvironment. Ten days and 6 weeks time points shown with DAPI. **(C)** GFP-OPCs survive and integrate within the GFAP+ injury milieu by 6 weeks. **(D)** GFP-OPCs arborize in the contusion injury microenvironment. O4 staining shown with DAPI. **(E,F)** Analysis of GFP-OPC and GFAP signal overlap at 6 weeks. GFAP and GFP signal is distinct with regions of overlap along STEM121 neural processes. Two examples are provided with zoom images. Asterisks depict GFP-OPC (green), GFAP (magenta), STEM121 (red), and signal overlap (white). R (rostral), C (caudal). Individual scale bars are provided.

We transplanted neurons without microenvironment modifying enzymes or encapsulation, but we included OPCs that are known to be susceptible to death in the injury microenvironment (Almad et al., 2011) and in addition have been shown to play a supportive role in recovery and myelination (Fan et al., 2017). OPCs were generated from hiPSCs expressing CAG-GFP by lentivirus transduction (Taylor et al., 2006) and were differentiated using a similar NMP protocol as for SMNs, except that DAPT was excluded during the first 5 days since this is used to favor generation of neurons. Validation and purification of OPCs derived through NMPs by this protocol (Olmsted et al., 2021) are described (Supplementary Figure 4) and include biomarkers OLIG2/PAX6, NKX2–2, A2B5, and O4, along with MACS separation *via* the O4 antigen. We combined OPCs with SMNs in a 1:2 ratio for transplantation into the contused C4-C5 SCI cavity as a mixed-cell suspension (~200,000 cell dose per animal; Figures 4–7). The 1:2 ratio was chosen as opposed to a 1:1 ratio of OPCs to SMNs since single oligodendrocytes can myelinate multiple neuronal axons (Goldman and Kuypers, 2015). Neural co-cultures were encapsulated in 150 μm neural ribbons for overnight shipping on ice from Albany, NY to Houston, TX (Olmsted et al., 2020, 2021) for recovery and transplantation studies. Post-shipping release of the SMN-OPCs for transplantation was accomplished by dissolving the alginate in sodium citrate. The cells were injected in rats in HBSS supplemented with 5 mM glucose and 20 ng/ml FGF/EGF.

We used a well-characterized, reproducible rat model of cervical SCI (Mondello et al., 2015) wherein right-sided C4-C5 hemi-contusion injuries were made using the electromagnetic spinal cord injury device (ESCID). Fifteen days post-injury, female Long-Evans rats (*N* = 6) received cervical intraspinal injections of cell suspensions (~200,000 cells delivered). Grafts were successfully placed in all six animals. Half of the animals were sacrificed 10 days post-transplantation (Figure 4), and the other half were sacrificed 6 weeks post-transplantation (Figures 5, 6). Features of the SMNs such as TUJ1 positivity and synaptic connections were clearly visible at 10 days (Figures 4D,E). SMNs and OPCs survived in all animals at 10 days and 6 weeks time points. At the extended 6 weeks time point. At the extended 6 weeks time point, grafted cells were again identified by immunohistochemistry that allowed discernment of host vs. transplanted human cells within the contusion cavity (GFAP, TUJ1, STEM121 comparisons in Figure 5) and neuronal projections with aligned fiber outgrowth into spared host tissue (GFAP, SYN1, STEM121 comparisons in Figure 6). Grafted STEM121+ SMNs co-localized with Synapsin 1 and expressed NeuN at the extended 6 weeks time point. Transplanted neurons were measured to project a maximum of 2.12 mm caudal and 1.52 mm rostral to the lesion epicenter. We performed Cavalieri analysis of the spinal cord, lesion, and grafts to determine graft volumetrics at 6 weeks post-transplantation (Figures 5E,F). Every five sections (40 μm thick), the area of the cord, lesion cavity, and graft (STEM121) were determined and volumes were calculated (*N* = 3 spinal cords analyzed). The volumes (mm^3^) for individual (*N* = 3) grafts and the corresponding residual lesions are provided in addition to the means. The volume of cell suspension injected was 2 μl (2 mm^3^). The graft volume remained substantial at 6 weeks, consistent with our immunohistochemistry staining that indicates good viability of neurons and OPCs without neuroprotection or additional supportive measures within the contused SCI microenvironment (Figures 5, 6).

Histological signs of OPC maturation over time are also evident comparing analyses at 10 days with 6 weeks (Figure 7) such as OPC ramification (Figures 7A–D). We also observed that in some frames the GFP-OPC signal appeared to be closely co-localized with that of GFAP (Figures 7E,F). Although the ability to count cells precisely is challenging in grafts, we quantified the total number of GFAP+ cells and determined the overlapping GFP-OPC/GFAP+ signals in 40 μm sections vs. GFP alone. The average estimated GFP-OPC population in three rats over five sections per animal is 50,570 ± 7,756 for GFP-OPCs where 6,717 ± 1,887 had GFP-OPC/GFAP+ co-localized signal (13.28%). GFP-OPC and GFAP signal overlap was only observed in subcellular compartments (neural processes) and not throughout entire cells. In most cases, distinct signals were identified in addition to regions of overlap (Figures 7E,F). Whether this result represents true transdifferentiation of OPCs to astrocytes, as has been reported in injury microenvironments (Valny et al., 2017), or instead the very close juxtaposition of these cells, cannot be easily discerned due to complex cell shape, disorganized tissue architecture at the injury site, and optical resolution restrictions for fine processes. Therefore, we cannot describe this as transdifferentiation based on the current data.

## Discussion

The recent advanced ability to generate regionally matched spinal cord neurons for SCI *via* NMP lineage and developmental cues emboldens a re-evaluation of these cells *in vitro* to model neural circuit formation (Nedelec and Martinez-Arias, 2021; Olmsted et al., 2021) and their *in vivo* evaluation in animal models. The ability to graft spinal cord matched neuronal-glial cell types by applying these principles *ex vivo* through hiPSC and NMP technology and has not been tested in postnatal mammals outside of the current work. We demonstrate survival *in vivo* in a rat hemicontusion model of SCI and provide analysis to benefit their use in future SCI grafts for long-term behavioral analyses. Transplanted SMNs were comprehensively characterized *in vitro*, functionally and by RNAseq, and demonstrated to exhibit inherent hallmarks of neuronal maturity including expression of Na_v_1.6 and K_v_1.2, axon initial segment formation, cholinergic and glutamatergic synaptogenesis with NMJ formation, and spontaneous as well as glutamate-responsive spiking activity. Our findings are consistent with and underscore the importance of homotypic patterning in cell therapeutics for the spinal cord (Dulin et al., 2018; Olmsted et al., 2020), as has emerged in foundational CNS studies for brain pathologies (Lund and Hauschka, 1976; Lindvall, 1989; Das, 1990; Lindvall et al., 1990; Dunnett, 1991). *In vivo*, we observed robust survival, rapid engraftment, neuronal fiber anisotropic projections, and histological evidence for synaptogenesis of unprotected SMNs and OPCs delivered in suspension into the challenging microenvironment of the hemicontusion injury cavity.

Neuronal grafting in the mammalian CNS has seen tremendous evolution, building from pre-stem cell studies with embryonic tissues across organisms (Bjorklund and Stenevi, 1984) to rapid innovation and discovery with stem cell technologies incorporating NSPCs (Thomson et al., 1998; Takahashi and Yamanaka, 2006). Trunk and spinal cord neural treatments with NSPCs could also adapt new NMP protocols used here vs. current methods that by default generate cells with obligate anterior telencephalic (Pankratz et al., 2007) rather than caudal trunk identity (Dulin et al., 2018; Kumamaru et al., 2018). The use of mature SMNs vs. trunk-directed NSPCs as a source for cell transplantation bypasses barriers inherent to multipotency and the potential for tumorigenicity.

Studies reveal that the refractory, immunoreactive SCI microenvironment generates inherent cell fate bias to differentiate along the astroglial lineage (Liu et al., 2019). Injury site modifiers applied with NSPCs, such as removal of inhibitory chondroitin sulfate proteoglycans (CSPGs) by use of the extracellular matrix glycosaminoglycan enzyme chondroitinase ABC (Bradbury and Burnside, 2019), may benefit grafts allowing NSPCs to differentiate towards neuronal outcomes and favor synaptogenesis and plasticity (Bradbury et al., 2002). We did not apply chondroitinase ABC in the current study. SMNs were co-transplanted with supporting NMP-derived OPCs purified by the OPC-specific biomarker O4. By immunohistochemistry we observed host GFAP+ astrocytes within the injury site, including a subset closely associated with the grafted GFP-OPCs. We evaluated merged fluorescent images to determine if transdifferentiation was occurring, as has been described previously in several studies (reviewed in Valny et al., 2017) in which OPCs can transdifferentiate to astrocytes in harsh CNS injury microenvironments. We determined that a significant fraction of co-signal overlap was due to merged signals from different planes as well as some very closely associated OPCs and GFAP+ staining cells along neuronal processes. It may still be possible that some transdifferentiation occurred at a very low frequency but will require future studies to validate such a transition in our graft-host contusion model.

An important step towards studying and fully achieving grafted complex circuit replacement in SCI is the development of greater reproducibility through improved methodologies. The use of homotypic, pre-defined neuronal and glial populations represents a new and exciting approach that allows immediate focus on circuitry regeneration. In the present study, we demonstrate that regionally appropriate stem cell-derived SMNs with maturing phenotype survive *in vivo* despite delivery into the challenging lesion cavity environment. The feasibility and reproducible methodologies for the delivery of pre-differentiated, functional cell types reported here constitute a critical step towards focused, targeted circuitry replacement in subacute lesions.

The findings of mature SMN engraftment as suspension-delivered cells are surprising and promising. Modern stem cell differentiation protocols are increasingly aided by newly revealed *in vivo* embryological principles for patterning spinal cord neural cells (Olmsted and Paluh, 2021) and are enabling alternative experimental strategies that combine encapsulation as a tool for pre-formation of transplantable neuronal networks (Olmsted et al., 2021). The *ex vivo* pre-differentiation of the cells allows comprehensive analysis by RNA-Seq, imaging of biomarkers, co-culture assays, and electrophysiology in order to ensure future reproducibility and to bypass NSPC-specific *in vivo* challenges of stochastic differentiation. Biomarker analysis of spinal cord sections in this study revealed robust survival of the homotypic neurons and OPCs, rapid engraftment within the host tissue at 10 days post-transplantation, and outgrowth at 6 weeks post-transplantation. Signs of graft cell maturation over time included OPC ramification, caudal axon anisotropic navigation, and increased co-localization with Synapsin within the spinal cord. The degree to which the electrophysiological profile of SMNs *in vitro* matches those *in vivo* is unknown. However, RNA-Seq revealed appropriate receptor profiles in the SMNs and functional *in vitro* assays confirmed neurotransmitter responsiveness. The robust survival and synaptic integration of the transplanted neurons *in vivo* is consistent with sufficient spinal cord matching.

The ability to fully interrogate SMNs and OPCs *in vivo* to restore circuitry and lost functional modalities will require larger cohorts and long-term circuitry evaluation with behavioral analyses. Our findings are expected to drive the field to develop advanced tools to address graft-host interconnectivity in circuitry reformation in real time, in addition to current methods of trans-synaptic labeling and transmission electron microscopy. As well, new strategies to boost OPC maturation and myelinating activity have been described through regulation of microRNAs (Fan et al., 2017). In our short-term study, the transplanted OPCs lost biomarkers for early progenitors but did not yet achieve myelinating activity at 6 weeks. Nevertheless, the cells remained in close contact with axons and became ramified. The precise role that grafted OPCs play in remyelination or support in regard to restored circuitry and functional behavior outcomes is unclear and not addressed this work. In a recent study, it was determined that myelinating activity of OPCs is not required for functional recovery (Duncan et al., 2018). We did not observe wrapping structures around axons in our GFP-OPCs, as were observed in Fan et al. (2017). Our approach may further benefit by incorporating additional defined cells such as ventral and/or dorsal spinal interneuron subtypes differentiated *in vitro* (Sagner and Briscoe, 2019; Zavvarian et al., 2020). Although we do not interrogate or quantify circuitry reformation, the functional pre-requisites of neuronal survival, synapse formation, and projection into the host are achieved. This work therefore demonstrates the exciting potential of transplanting pre-differentiated neurons within a lesion cavity to advance regionally specified circuit reconstruction and repair studies, as well as injury customization towards improved *in vivo* SCI therapeutic outcomes.

## Data Availability Statement

The datasets presented in this study can be found in online repositories. The names of the repository/repositories and accession number(s) can be found below: https://www.ncbi.nlm.nih.gov/geo/query/acc.cgi?acc=GSE178600.

## Ethics Statement

The animal study was reviewed and approved by the Houston Methodist Research Institute IACUC.

## Author Contributions

JP and ZO designed human stem cell experiments and wrote the manuscript with input from all co-authors. ZO compiled figures and performed hiPSC neural differentiation and analysis, *in vitro* imaging, mRNA sample preparation, and MEA experiments. BM assisted with RNA-Seq samples, analysis, and bioinformatics. PH and CS designed and conducted rat experiments for *in vivo* grafting. CS performed histology, data acquisition and analysis. The F3.5.2 hiPSC line was derived previously in collaboration with JC, who also provided key input for experiments and analysis. All authors contributed to the article and approved the submitted version.

## Conflict of Interest

The authors declare that the research was conducted in the absence of any commercial or financial relationships that could be construed as a potential conflict of interest.

## Publisher’s Note

All claims expressed in this article are solely those of the authors and do not necessarily represent those of their affiliated organizations, or those of the publisher, the editors and the reviewers. Any product that may be evaluated in this article, or claim that may be made by its manufacturer, is not guaranteed or endorsed by the publisher.
