# Supplementary material for: Fully Characterized Mature Human iPS- and NMP-Derived Motor Neurons Thrive Without Neuroprotection in the Spinal Contusion Cavity
Source: Front Cell Neurosci. 2022 Jan 3;15:725195. doi: 10.3389/fncel.2021.725195 (PMC8762343; doi:10.3389/fncel.2021.725195)
Supplement: Supplementary file 1 [file Data_Sheet_1.PDF]

SUPPLEMENTAL INFORMATION

Fully characterized mature human iPS- and NMP-derived motor neurons thrive without neuroprotection in the spinal contusion cavity

Zachary T. Olmsted[1], Cinzia Stigliano[2], Brandon Marzullo[3], Jose Cibelli[4], Philip J. Horner[2], and Janet L. Paluh[1*]

***Corresponding author:** Dr. Janet Paluh
State University of New York Polytechnic Institute CNSE
4424 NanoFab East
257 Fuller Road, Albany, NY 12203
Phone: (518) 956-7047
Fax: (518) 437-8687
Email: jpaluh@sunypoly.edu

Figure S1

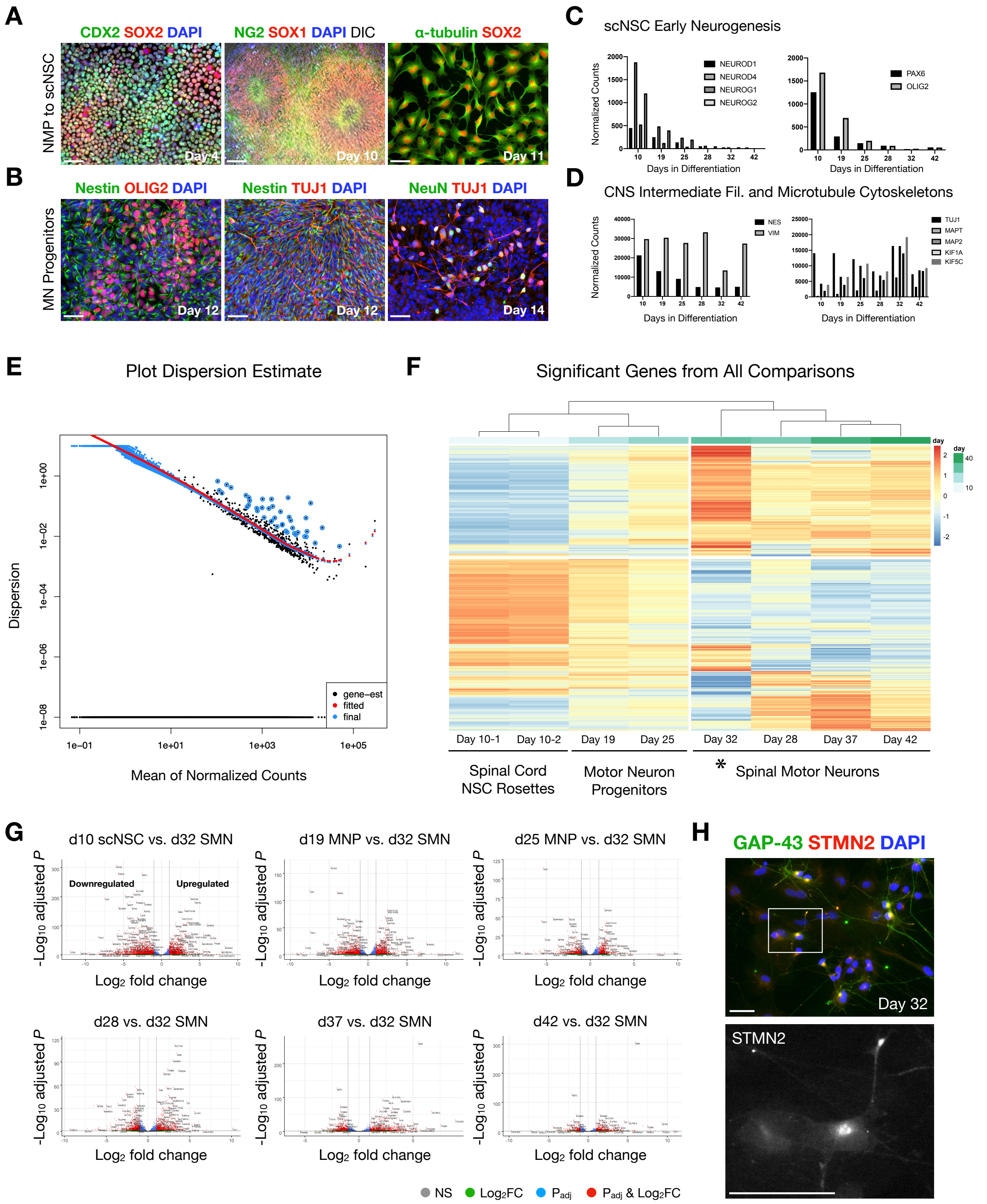

A
CDX2 SOX2 DAPI
NG2 SOX1 DAPI DIC
α-tubulin SOX2
NMP to scNSC
Day 4
Day 10
Day 11
B
Nestin OLIG2 DAPI
Nestin TUJ1 DAPI
NeuN TUJ1 DAPI
MN Progenitors
Day 12
Day 12
Day 14
C
scNSC Early Neurogenesis
Normalized Counts
NEUROD1
NEUROD4
NEUROG1
NEUROG2
PAX6
OLIG2
Days in Differentiation
D
CNS Intermediate Fil. and Microtubule Cytoskeletons
NES
VIM
TUJ1
MAPT
MAP2
KIF1A
KIF5C
E
Plot Dispersion Estimate
Dispersion
gene-est
fitted
final
Mean of Normalized Counts
F
Significant Genes from All Comparisons
day
Day 10-1
Day 10-2
Day 19
Day 25
Day 32
Day 28
Day 37
Day 42
Spinal Cord NSC Rosettes
Motor Neuron Progenitors
* Spinal Motor Neurons
G
d10 scNSC vs. d32 SMN
d19 MNP vs. d32 SMN
d25 MNP vs. d32 SMN
d28 vs. d32 SMN
d37 vs. d32 SMN
d42 vs. d32 SMN
Downregulated
Upregulated
-Log10 adjusted P
Log2 fold change
NS
Log2FC
Padj
Padj & Log2FC
H
GAP-43 STMN2 DAPI
Day 32
STMN2

Figure S1. RNA-Seq bioinformatics analyses of scNSC neuronal differentiation. A, CDX-2/SOX2 NMPs generate NG2/SOX1 scNSC rosettes that can be reseeded as spinal cord neural progenitor cell adherent cultures (α-tubulin/SOX2). **B**, Caudo-ventral patterning of scNSCs yields motor neuron progenitors (MNPs) expressing biomarkers Nestin, OLIG2, TUJ1 and NeuN. **C**, Histogram of normalized counts by RNA-Seq reveals progressive downregulation of transcription factors involved in neurogenesis (left) and motor neuron-specific patterning (right) from scNSCs to a differentiated state. **D**, Normalized counts of CNS-related intermediate filament (Nestin, Vimentin; left) and microtubule cytoskeleton proteins (right) by RNA-Seq. **E**, Plot Dispersion Estimate of RNA-Seq samples. **F**, Heatmap of significant genes from all comparisons for SMN differentiation from scNSC stage. Day 32 (*) constitutes the most suitable stage for our SMNs on the basis of electrophysiological function compared to a global analysis of SMN and neuron-related RNA transcript expression. This time point was additionally used for *in vivo* transplantation studies. **G**, Volcano plots of differentiation time points with reference to day 32 SMNs. **H**, Top: IF of differentially expressed GAP-43 and STMN2 in day 32 adherent SMNs. Bottom: STMN2 characteristic perinuclear and growth cone localization (Klim et al., 2019). Scale bars are 50 μm.

Figure S2

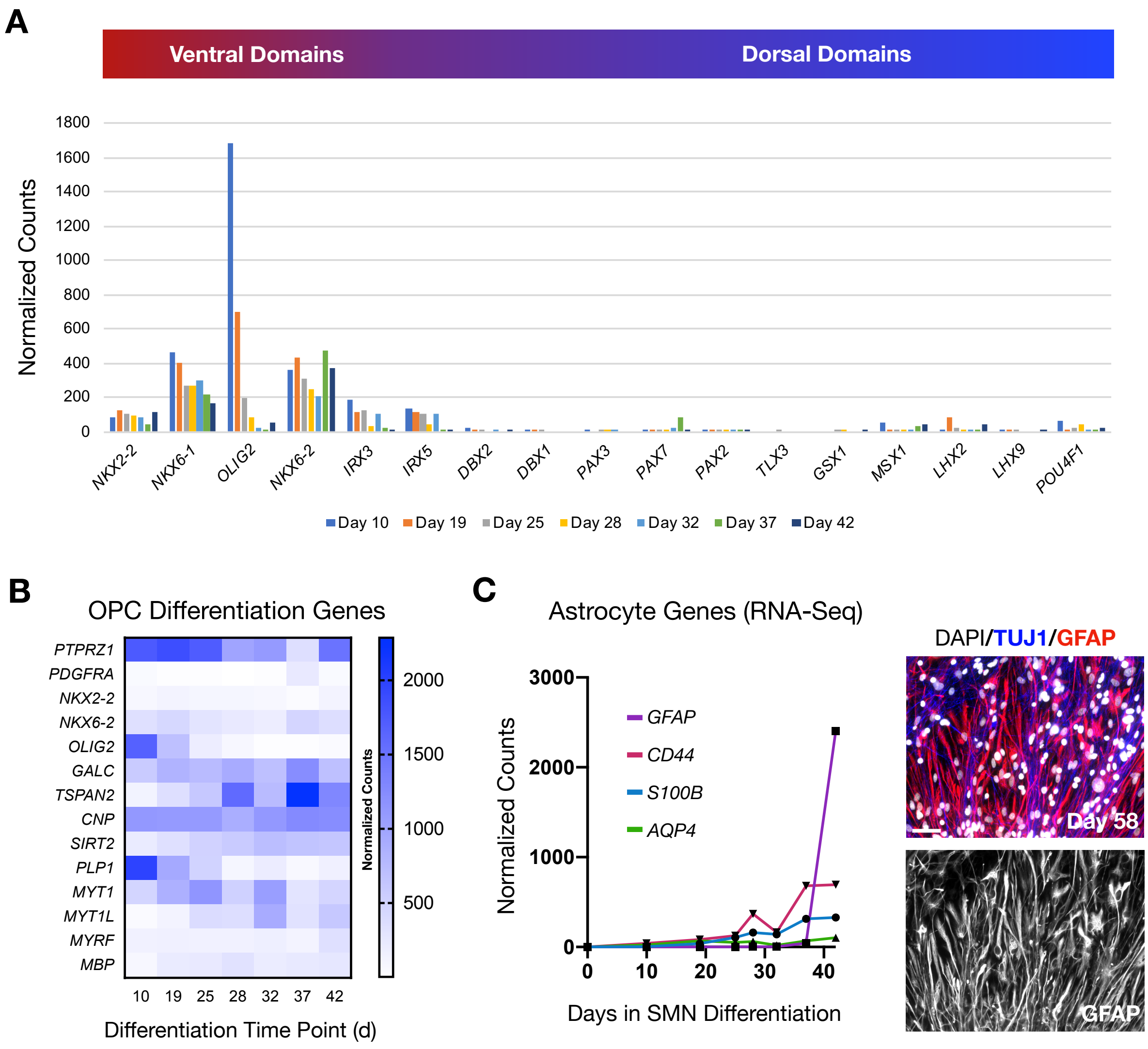
A
Ventral Domains
Dorsal Domains
Normalized Counts
1800
1600
1400
1200
1000
800
600
400
200
0
NKX2-2
NKX6-1
OLIG2
NKX6-2
IRX3
IRX5
DBX2
DBX1
PAX3
PAX7
PAX2
TLX3
GSX1
MSX1
LHX2
LHX9
POU4F1
Day 10
Day 19
Day 25
Day 28
Day 32
Day 37
Day 42
B
OPC Differentiation Genes
PTPRZ1
PDGFRA
NKX2-2
NKX6-2
OLIG2
GALC
TSPAN2
CNP
SIRT2
PLP1
MYT1
MYT1L
MYRF
MBP
10
19
25
28
32
37
42
Normalized Counts
2000
1500
1000
500
Differentiation Time Point (d)
C
Astrocyte Genes (RNA-Seq)
Normalized Counts
3000
2000
1000
0
GFAP
CD44
S100B
AQP4
0
10
20
30
40
Days in SMN Differentiation
DAPI/TUJ1/GFAP
Day 58
GFAP

Figure S2. Quantifying sub-populations of interneurons and glia that emerge in SMN cultures. A, Histogram of RNA-Seq normalized counts for ventral (red) and dorsal (blue) spinal cord domain transcription factors over time in differentiating SMN cultures at day 58 (Sagner and Briscoe, 2019). **B**, Heatmap of differentiation genes for OPCs that originate from the motor neuron progenitor ventral domain in differentiating SMN cultures. **C**, RNA-Seq normalized counts over time for astrocyte biomarkers GFAP, CD44, S100B and AQP4 in differentiating SMN cultures (left). A wave of gliogenesis occurs at late stage timepoints; GFAP/TUJ1 mixed culture (day 58). Cells are counterstained with DAPI (right).

Figure S3

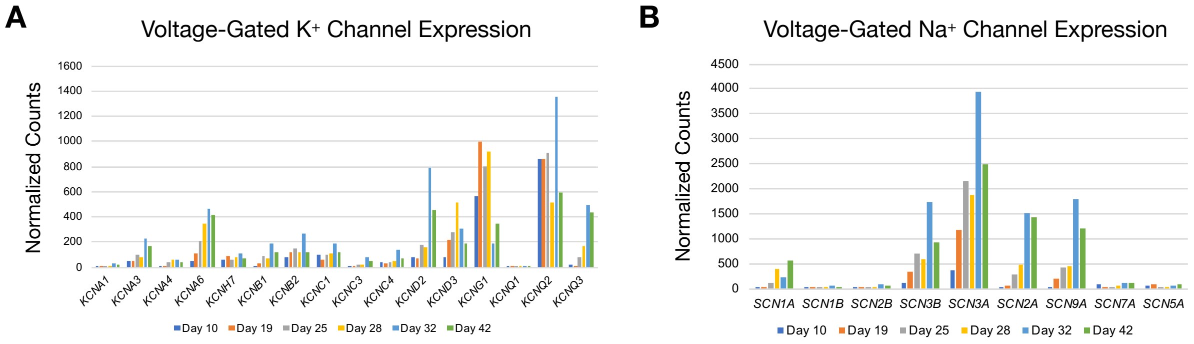
A
Voltage-Gated K+ Channel Expression
Normalized Counts
0
200
400
600
800
1000
1200
1400
1600
KCNA1
KCNA3
KCNA4
KCNA6
KCNH7
KCNB1
KCNB2
KCNC1
KCNC3
KCNC4
KCND2
KCND3
KCNG1
KCNQ1
KCNQ2
KCNQ3
Day 10
Day 19
Day 25
Day 28
Day 32
Day 42
B
Voltage-Gated Na+ Channel Expression
Normalized Counts
0
500
1000
1500
2000
2500
3000
3500
4000
4500
SCN1A
SCN1B
SCN2B
SCN3B
SCN3A
SCN2A
SCN9A
SCN7A
SCN5A
Day 10
Day 19
Day 25
Day 28
Day 32
Day 42

Figure S3. Ion channels and synaptic phenotypes in differentiating SMNs. Voltage-gated K^+ (**A**) and Na^+ (**B**) channel expression by normalized counts in differentiating SMN cultures.

Figure S4

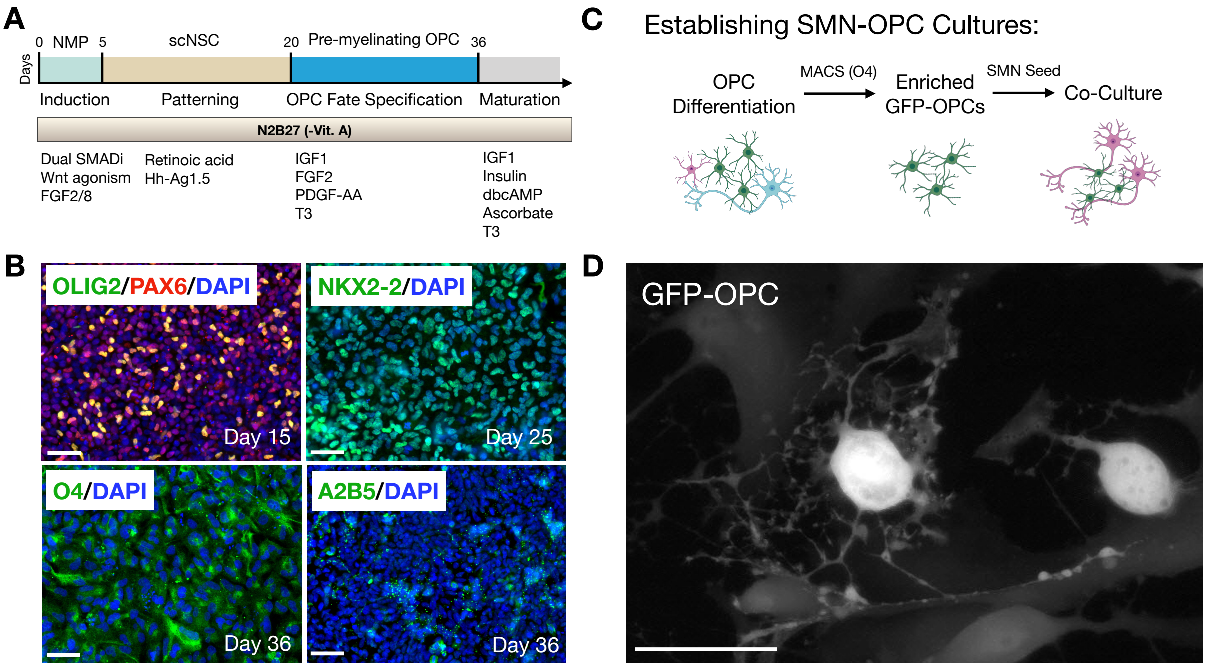
A
0
NMP
5
scNSC
20
Pre-myelinating OPC
36
Days
Induction
Patterning
OPC Fate Specification
Maturation
N2B27 (-Vit. A)
Dual SMADi
Wnt agonism
FGF2/8
Retinoic acid
Hh-Ag1.5
IGF1
FGF2
PDGF-AA
T3
IGF1
Insulin
dbcAMP
Ascorbate
T3
B
OLIG2/PAX6/DAPI
Day 15
NKX2-2/DAPI
Day 25
O4/DAPI
Day 36
A2B5/DAPI
Day 36
C
Establishing SMN-OPC Cultures:
OPC
Differentiation
MACS (O4)
Enriched
GFP-OPCs
SMN Seed
Co-Culture
D
GFP-OPC

Figure S4. Differentiation of hiPSCs to oligodendrocyte progenitor cells. A, Overview of OPC differentiation. **B**, IF characterization of differentiating OPCs. OLIG2/PAX6, NKX2-2, O4 and A2B5 biomarkers shown with DAPI. **C**, Schematic of OPC separation by MACS using the O4 antigen and subsequent co-culture with SMNs. **D**, GFP-OPC after separation. Scale bars are 50 μm.

Supplemental Tables

Table S1. Antibodies Used *In Vitro*

Target	Company; Cat#	Species; Clone
AnkG	Antibodies Inc.; 75-147	Mouse mAb IgG_{2B}; N106/65
α-tubulin	Invitrogen; 62204; RRID: AB_1965960	Mouse mAb IgG_1; DM1A
β4-Spectrin	Antibodies Inc.; 73-376; RRID: AB_2315816	Mouse mAb IgG_1; N393/2
CDX2	R&D Systems; MAB3665	Mouse mAb IgG_1; 963809
ChAT	R&D Systems; MAB3447	Mouse mAb IgG_1; #334008
Desmin	R&D Systems; AF3844; RRID: AB_2092419	Goat pAb IgG
FOXP1	Santa Cruz Biotechnologies; sc-398811	Mouse mAb IgG_1; A-2
GAP-43	Encor; MCA-5E8; RRID: AB_2572287	Mouse mAb IgG_1; GAP43
Gephyrin	Antibodies Inc.; 75-444; RRID: AB_2636852	Mouse mAb IgG_{2A}; L106/93
GFAP	R&D Systems; MAB2594	Mouse mAb IgG_1; #273807
GluN2A (*GRIN2A*)	Antibodies Inc.; 75-288; RRID: AB_2120479	Mouse mAb IgG_{2A}; N355/1
GluR1 (*GRIA1*)	Antibodies Inc.; 75-327; RRID: AB_2315840	Mouse mAb IgG_1; N355/1
HB9 (*MNR2/MNX1*)	DSHB; 81.5C10; RRID: AB_2145209	Mouse mAb IgG_{1K}; 81.5C10
Hox-C6	Santa Cruz Biotechnologies; sc-376330	Mouse mAb IgG_{2A}; B-7
ISL1	Millipore Sigma; HPA057416; AB_2683431	Rabbit pAb IgG
ISL1&2	DSHB; 39.4D5; RRID: AB_2314683	Mouse mAb IgG_{2B}; 39.4D5
K_v1.2 (*KCNA2*)	Novus Biologicals; NBP1-42802SS; RRID: AB_2128586	Rabbit pAb IgG
LHX3	Santa Cruz Biotechnologies; sc-293411	Mouse mAb IgG_{2A}; 2C10
MAP2	Abcam; ab32454; RRID: AB_776174	Rabbit pAb IgG
Myogenin	R&D Systems; MAB6686	Mouse mAb IgG_1; #671038
Myosin Heavy Chain	R&D Systems; MAB4470; RRID: AB_1293549	Mouse mAb IgG_{2B}; #MF20
NCAM-1	R&D Systems; AF2408; RRID: AB_442152	Goat pAb IgG
Nestin	R&D Systems; MAB1259; RRID: AB_2282664	Mouse mAb IgG_1; 196908
NeuN	Millipore Sigma; MAB377; AB_2298772	Mouse mAb IgG_1; A60
NG2 (*CSPG4*)	eBioscience; 14-6504-80	Mouse mAb IgG_{2A}; 9.2.27
Nkx-2.2	DSHB; 74.5A5; RRID: AB_531794	Mouse mAb IgG_{2B}; 74.5A5
Nkx-6.1	DSHB; F55A12; RRID: AB_532379	Mouse mAb IgG_1; F55A12
OLIG2	R&D Systems; AF2418; RRID_2157554	Goat pAb IgG
O4	R&D Systems; MAB1326; RRID: AB_357617	Mouse mAb IgM; #O4
O4	Chemicon; MAB345; RRID: AB_94872	Mouse mAb IgM; 81
PAX6	DSHB; PAX6; RRID: AB_528427	Mouse mAb IgG_1; PAX6
Pan-Cadherin	Abcam; ab6528; AB_305544	Mouse mAb IgG_1; CH-19
PE-O4	R&D Systems; FAB1326P; RRID: AB_664169	Mouse mAb IgM; #O4
Peripherin	Santa Cruz Biotechnologies; sc-377093	Mouse mAb IgG_{2A}; A-3
PSD-95 (*DLG4*)	Abcam; ab18258; RRID: AB_2292909	Rabbit pAb IgG
RALDH2 (*ALDH1A2*)	Millipore Sigma; ABN420	Rabbit pAb IgG
SMI311R	BioLegend; 837801; RRID: AB_2565383	Mouse mAb IgG/IgM cocktail; SMI311R
SMI312	BioLegend; 837904; RRID: AB_2566782	Mouse mAb IgG_K/IgM_K cocktail; SMI312
SOX1	R&D Systems; AF3369; RRID: AB_2239879	Goat pAb IgG
SOX2	R&D Systems; MAB2018; RRID: AB_358009	Mouse mAb IgG_{2A}; 245610
SOX2	R&D Systems; AF2018; AB_355110	Goat pAb IgG
STMN2	Novus Biologicals; NBP149461; RRID: AB_10011569	Rabbit pAb IgG
Synapsin 1 (*SYN*)	Millipore Sigma; AB1543; RRID: AB_2200400	Rabbit pAb IgG (serum)
TUJ1 (β-III-tubulin, *TUBB3*)	BioLegend; 802001; RRID: AB_2564645	Rabbit pAb IgG; Poly18020
TUJ1 (β-III-tubulin, *TUBB3*)	Abcam; ab7751; RRID: AB_306045	Mouse mAb IgG; ab7751
VGF	Abcam; ab74140; RRID: AB_1524551	Rabbit pAb IgG

VGlut1	Antibodies Inc.; 75-066; RRID: AB_2187693	Mouse mAb IgG_1; N28/9

Table S2. RNA-Seq Samples

Day in differentiation	Stage	Purification	Differentiation	Wells pooled (12w plate)	Comments
10	scNSC rosettes	N/A	A	3	Biol. replicate
10	scNSC rosettes	N/A	B	3	Biol. replicate
19	MNP	N/A	A	3	CVPM[a] at day 5
25	MNP	N/A	A	3	CVPM at day 5
28	SMN	N/A	A	3	TDM[b] at day 25
32	SMN	NS (day 28)	A	3	Plated day 29
37	SMN	NS (day 28)	A	3	Plated day 29
42	SMN	NS (day 28)	A	3	Plated day 29

[a] Caudo-Ventral Patterning Medium (N2B27 + RA, Hh-Ag1.5)

[b] Terminal Differentiation Medium (N2B27 + BDNF, GDNF, dbcAMP)
